# Cathartocytosis: Jettisoning of cellular material during reprogramming of differentiated cells

**DOI:** 10.1016/j.celrep.2025.116070

**Published:** 2025-07-30

**Authors:** Jeffrey W. Brown, Xiaobo Lin, Gabriel Anthony Nicolazzi, Xuemei Liu, Thanh Nguyen, Megan D. Radyk, Joseph Burclaff, Jason C. Mills

**Affiliations:** 1 Division of Gastroenterology, Department of Medicine, Washington University in St. Louis, School of Medicine, St. Louis, MO, USA; 2 Section of Gastroenterology & Hepatology, Departments of Medicine, Pathology & Immunology, and Molecular and Cellular Biology, Baylor College of Medicine, Houston, TX 77030, USA; 3 Cancer and Cell Biology Graduate Program, Graduate School of Biomedical Sciences, Baylor College of Medicine, Houston, TX, USA; 4 Present address: Department of Molecular & Integrative Physiology, University of Michigan, Ann Arbor, MI, USA; 5 Present address: Joint Department of Biomedical Engineering, University of North Carolina at Chapel Hill and North Carolina State University, Chapel Hill, NC 27599, USA; 6 Lead contact

## Abstract

Injury causes differentiated cells to undergo massive reprogramming to become proliferative and repair tissue via paligenosis. Gastric chief cells use paligenosis to reprogram into progenitor-like spasmolytic-polypeptide-expressing metaplasia (SPEM) cells. Stage 1 of paligenosis is the downscaling of mature cell architecture via a process involving lysosomes. Here, we notice that sulfated glycoproteins are not only digested during paligenosis but also excreted into the gland. Various genetic and pharmacological approaches show that endoplasmic reticulum membranes and secretory granule cargo are also excreted and that the process proceeds in parallel with but is mechanistically independent of autophagy. Three-dimensional light and electron microscopy demonstrated that excretion occurs via unique, complex, multi-chambered invaginations of the apical plasma membrane. As this lysosome-independent cell cleansing process does not seem to have been priorly described, we termed it “cathartocytosis.” Cathartocytosis allows a cell to rapidly eject excess material without waiting for autophagic and lysosomal digestion, providing for efficient cellular downscaling.

## In brief

Cellular plasticity, which involves massive changes to a cell’s architecture and organellar organization, can be harnessed following injury to repair damaged tissue. Brown et al. describe cathartocytosis, a process that large, differentiated cells can use to rapidly downscale their cellular machinery.

## Graphical Abstract

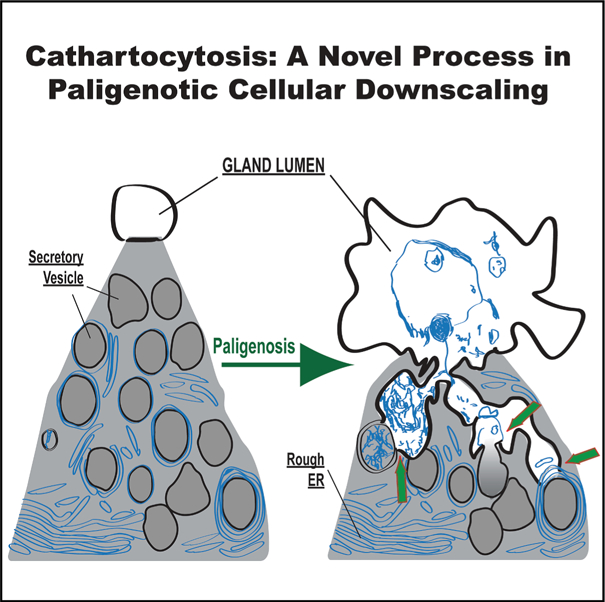

## INTRODUCTION

When an injury causes the loss of mature cells in tissues with constitutively active, multipotent stem cells, the tissue can be repaired by increasing stem cell proliferation and differentiation, thereby replacing both the lost cell mass and maintaining the stem cell pool.^1^ However, when stem cells themselves are damaged or when tissues lack dedicated stem cells, the burden of repair falls on mature, differentiated cells.^2^ As a consequence, differentiated cells have evolved a capacity to respond to injury by reprogramming to a downscaled proliferative phenotype to regenerate damaged cells, replace dead cells, and maintain barrier function.^3^ It has been proposed that an evolutionarily conserved program executes the reprogramming of mature cells to cycling progenitors. Specifically, our group has shown that following injury, post-mitotic differentiated cells can reprogram into proliferative, immature-appearing cells through a series of three stepwise stages in a conserved process known as paligenosis.^4^

Briefly, during the response to injury, a cell undergoing paligenosis tunes down mTORC1 activity and massively upregulates autodegradative machinery, characterized by expanded lysosome activity and induction of autophagy (stage 1).^5^ During this phase of cellular downscaling, mature cellular machinery is dismantled and removed, becoming superfluous as the cell transitions to a proliferative progenitor state for tissue repair. In stage 2 of paligenosis, autophagy subsides, and mTORC1 activity increases coincident with the expression of metaplasia-related, progenitor-cell proteins like SOX9.^6^ Next, cells can proceed into the cell cycle (stage 3) if they successfully suppress p53 and its licensing function.^7,8^ The reprogrammed chief cells are known as spasmolytic-polypeptide-expressing metaplasia (SPEM) cells,^9–13^ and the organ-level metaplastic transformation is referred to as pyloric or pseudopyloric metaplasia due to the antralized appearance of the corpus with light microscopy.^14,15^ One feature of metaplasia and tumorigenesis in the stomach (and also the pancreas and esophagus) is the induced expression and secretion of acidic (sialylated and sulfated) glycoproteins.^16–20^ More recently, we have shown that extracellular (presumably secreted) sulfated glycoproteins can be used as clinical biomarkers to detect and stratify risk for these cellular transformations.^20–23^

Here, we track the subcellular distribution and movement of both the zymogenic granules and the endoplasmic reticulum (ER) as the gastric chief cells downscale their cellular machinery en route to metaplasia. We show that these two organellar compartments, which constitute the vast majority of the gastric chief cell cytoplasm, are—as might have been predicted based on previous work—routed to autophagic degradation in RAB7^+^/LAMP2^+^ late endosomes/lysosomes (LEs/Lys). However, we also find, surprisingly, that this cellular material is excreted from the cell in a process that is mechanistically independent of canonical autophagy. Using focused ion beam scanning electron microscopy (FIB-SEM), we structurally characterize the secretory apparatus for the excretion process and find that it occurs via phagophore-shaped apical membrane invaginations. We propose the term cathartocytosis (in Greek, cellular cleansing) to describe this excretion process and believe it is at least partially responsible for the delivery of sulfated mucins and extracellular vesicles into the lumen of the gastric gland. As these sulfated mucins can be tumorigenesis biomarkers, cathartocytosis may be potentially useful for diagnosis and prognosis.

## RESULTS

### Distribution of sulfated glycosylation epitopes during paligenosis

We first wanted to better characterize our tools for assessing glycans in metaplasia, focusing on the antibody Das-1, which has been used to stage oncogenic transformation in the foregut (i.e., stomach, esophagus, and pancreas) and which we have previously shown recognizes 3ʹ-sulfo-Le^A/C^ (Figure S1).^20,24^ Figure S2 shows that the sulfated glycosylation epitopes recognized by Das-1 are on branched glycan trees that are O-linked and bound to proteins but not lipids. Evidence for this is that only NaOH (which strips O-glycans) eliminated Das-1 reactivity in western blot and paraffin-embedded, fixed tissue, whereas glycosidases that cleave N-linked or unbranched O-linked glycans did not. Also, as expected, neuraminidase (which cleaves sialylated, not sulfated, glycans) had no effect on the Das-1 signal. Moreover, neither chloroform extraction of lipids in solution nor xylene treatment of paraffin sections extracted the Das-1 signal, indicating that the Das-1 epitope was not attached to lipids.

**Figure 1.**
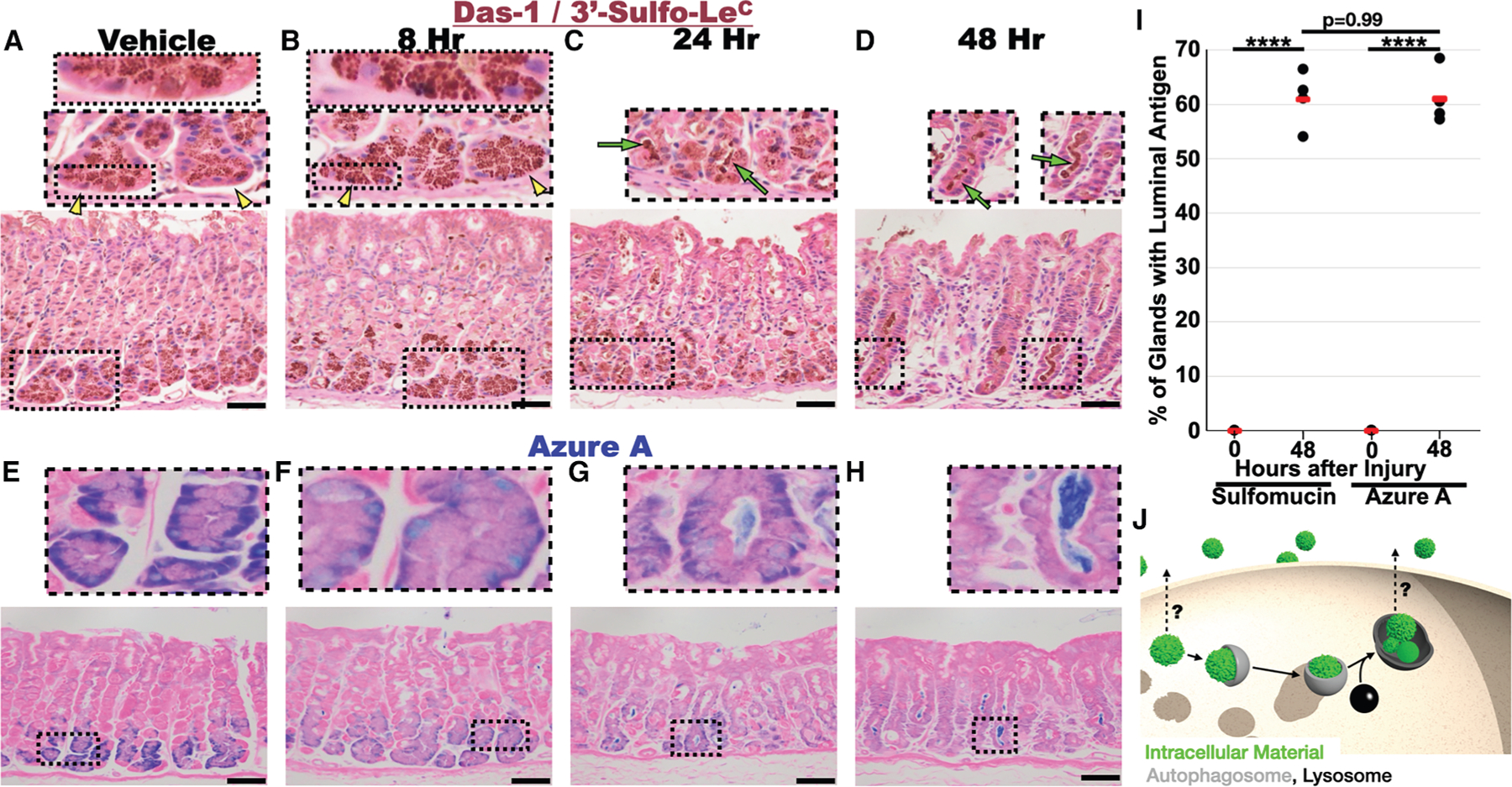
Cellular material is excreted during paligenosis (A–H) Micrographs of the mouse stomach after treatment with vehicle (A and E) and after injury (B and F: 8 h, C and G: 24 h, and D and H; 72 h following the first injection of high-dose tamoxifen). (A)–(D) are immunostained with Das-1, which recognizes the glycosylation epitope 3ʹ-sulfo-Le^A/C 24^ and (E)–(H) are stained with azure A and eosin. (I) Quantification of secreted luminal antigens 48 h after injury. Mean of all mice (*n =* 4–5) per condition depending on experiment. Pairwise significance was calculated with t test. (J) Schematic depicting potential trajectories for secreted of generic cytoplasmic organelles (green: intracellular material-like granule, ER, mitochondria, etc.). Gray: classical autophagy; black: lysosome. Yellow arrowheads demonstrate the loss of apical compartmentalization of the zymogenic granules 8 h after injury, relative to vehicle. The secretory granules are apical to the nucleus in (A) but also basal to the nucleus in (B). Green arrows identify secreted sulfated glycoproteins. Scale bars: 50 μm.

We next sought to determine how sulfated glycoprotein expression changes during injury-induced metaplasia. The injection of high doses of tamoxifen causes a characteristic, reversible injury pattern in the gastric epithelium marked by the loss of acid-pumping parietal cells and the reprogramming of chief cells into a metaplastic lineage. The effects of tamoxifen are independent of estrogen modulation and occur in both sexes.^25–27^ The conversion of the large secretory architecture of a chief cell into a downscaled, mitotic SPEM cell occurs by a stereotypical sequence of molecular-cellular events known as paligenosis^2,4,5^ and can be completed in as little as 48 h from the first injection of tamoxifen (note the statistically significant reduction in cell size shown in Figure S3). Das-1 immunohistochemistry (IHC) showed that, unlike what had previously been documented in the normal *human* stomach,^24^ homeostatic murine chief cells express sulfated glycoproteins (Figures 1A and S2I). The subcellular distribution was within cytoplasmic vesicles apical to the nucleus, as can be seen by the localization of Das-1 with the chief cell secretory vesicle cargo gastric intrinsic factor (GIF) (Figures S2H–S2J).

The first stage of paligenosis lasts up to 24 h and involves massive upregulation of autophagic and lysosomal structures as cells remodel their secretory apparatus. We noted some relocalization basally (i.e., closer to the nucleus) of Das-1-labeled vesicles by 8 h (Figure 1B); however, by 24 h, a time point when injured chief cells are completing the lysosomal/autophagic degradation stage and the cell has shrunk considerably (Figure S3), we observed dramatic reorganization of Das-1-labeled structures. Namely, sulfomucins began to appear in the lumen of the gland apical to chief cells (Figure 1C). By 48 h, a time point when most cells have converted to the proliferative SPEM phenotype, the sulfated glycoproteins were no longer detected in the downscaled chief cells. Instead, they could be found as cast-like structures throughout the lumens of gastric glands (Figure 1D).

### Extrusion of cellular material and organelles during downscaling in paligenosis

**Figure 2.**
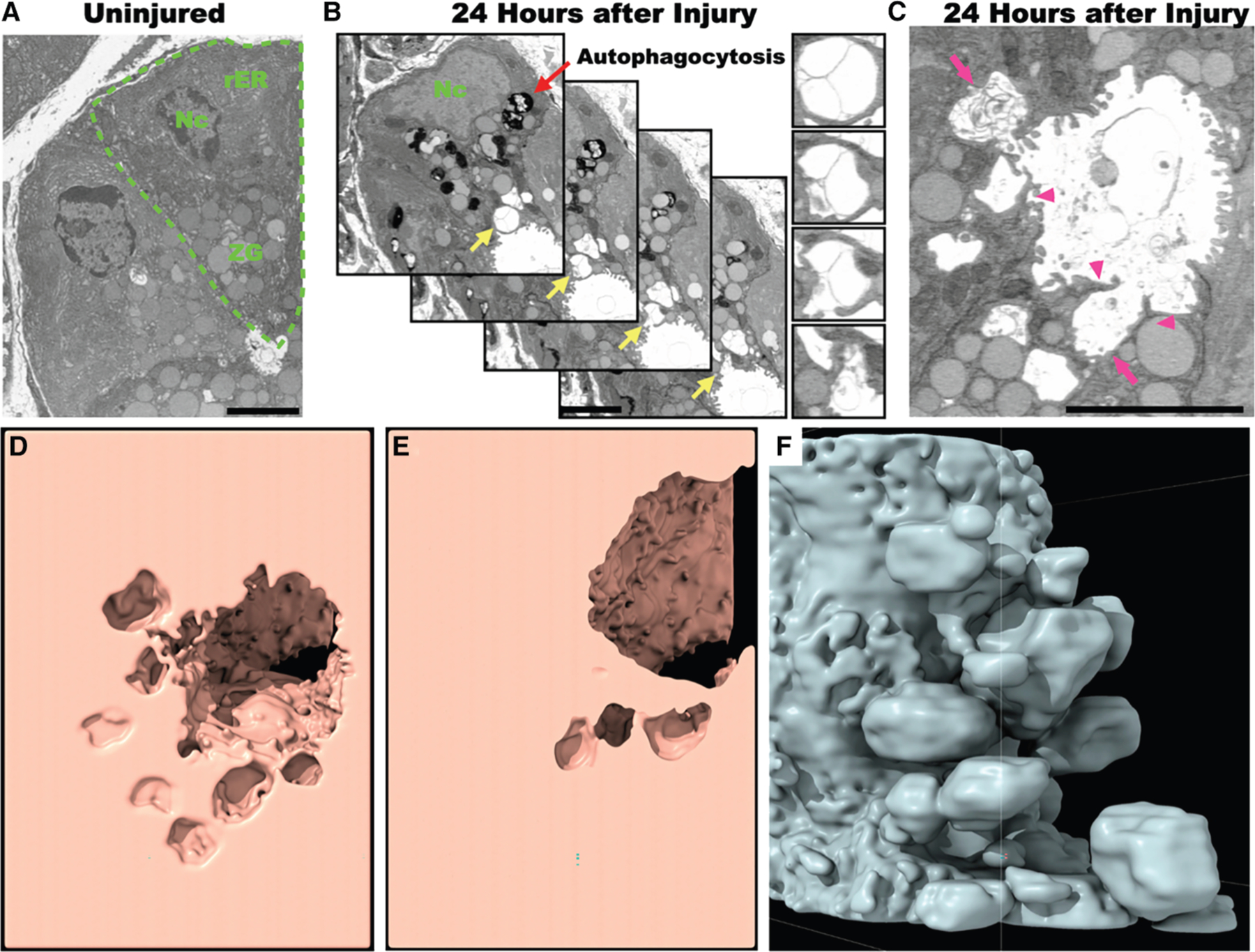
Focused ion beam scanning electron microscopy demonstrates apical membrane deformation and secreted vesicular material 24 h after injury (A) Vehicle-treated mouse stomach displaying two mature gastric chief cells in two-dimensional electron microscopic section. The boundaries and organellar compartments of one of these cells are annotated in green (Nc, nucleus; rER, rough endoplasmic reticulum; ZG, zymogenic granules). (B) Four sequential focused ion beam scanning electron microscopy (FIB-SEM) micrographs depicting excretion of large, electron-lucent extracellular vesicles and membranous material (red arrow: lysosomes and various stages of autophagosomes; yellow arrows highlights apical plasma membrane shown in inset at right). (C) Another section from FIB-SEM showing membrane material being extruded apically (top arrow) as well as the gland lumen. Note the distorted projections of apical plasma membrane into lumen (magenta arrowheads) and pit-like invaginations of apical plasma membrane (magenta arrowheads). (D–F) Three-dimensional reconstructions produced by stacking FIB-SEM sections. (D and E) Two slices through a three-dimensional reconstruction of how the gland lumen invaginates into an interconnected network of saccules and channels made by the plasma membrane of a paligenotic chief cell. The entire clipping series is available in Video S1. (F) Similar view but with convex reconstruction instead of concave. Note that the paligenotic chief cell demonstrates a series of communicating chambers invaginating into apical cytoplasm. A 360° rotational series of this model is provided as Video S2. Scale bars: 4 μm.

To better understand how sulfated glycoproteins were relocalizing during paligenosis, we established some tools to follow organellar changes. A differentiated gastric chief cell is characterized by a basal, globoid nucleus surrounded by an abundant, tightly packed lamellar rough ER (rER) with an apical collection of large secretory granules (Figures 1A, 1E, 2A, S4, and S5). We followed rER downscaling during these same first 24 h using FIB-SEM, the histological stain azure A (Figures 1E–1H and S4), and two different antibodies against the ER-resident proteins: (1) peptide protein disulfide isomerase (PDI) and (2) translocon-associated protein alpha subunit (TRAP alpha) (Figure S5).

Azure A is most commonly used in thin liquid chromatography to identify sulfatides (sulfated glycolipids)^28^ as well as sulfated glycosylaminoglycans (GAGs).^29^ Though we do not know the exact macromolecules bound by azure A in the gastric epithelium, we hypothesize that they are sulfated GAGs rather than sulfated glycolipids because (1) the tissue was processed with xylene, which removes lipids, and (2) antigen retrieval results in the loss of azure A material (unlike proteins, GAGs are not cross-linked with formalin^30^). We also observed staining of nuclei by azure A, likely due to association with acidic deoxyribonucleotides; however, nuclei had a distinctly lighter, royal blue staining compared to the presumptive ER (Figures 1E–1H and S4).

All techniques yielded consistent patterns at homeostasis: dense staining in the base of the cell that became more muted apically where zymogenic granules filled most of the cytoplasm, appearing as punched-out holes in a more lattice-like ER pattern (Figures 1, 2A, S4, and S5). Following injury, the dense basal staining of homeostatic cells was first consistently and notably decreased by 8 h (Figures 1F, S4B, and S4F). Analogous to the sulfated mucins we tracked using Das-1, extrusion or secretion of azure A-stained material occurred at 24 h, and the gland lumens were full of ER markers by 48–72 h (Figures 1G, 1H, S4, and S5). By 48 h, ∼60% of gastric glands had luminal azure A and Das-1 antigen (Figure 1I). As we observed paligenotic cells extruding or secreting an organelle (namely, the ER) along with sulfated glycoproteins, the phenomenon was clearly different from normal exocytotic secretion in chief cells. Typically, cargo (digestive enzymes) is elaborated extracellularly by merocrine secretion, in which the secretory vesicle fuses with the apical membrane and only cargo is released into the lumen, with all membranes being conserved.

### Ultrastructural changes in the apical membrane revealed by FIB-SEM

**Figure 3.**
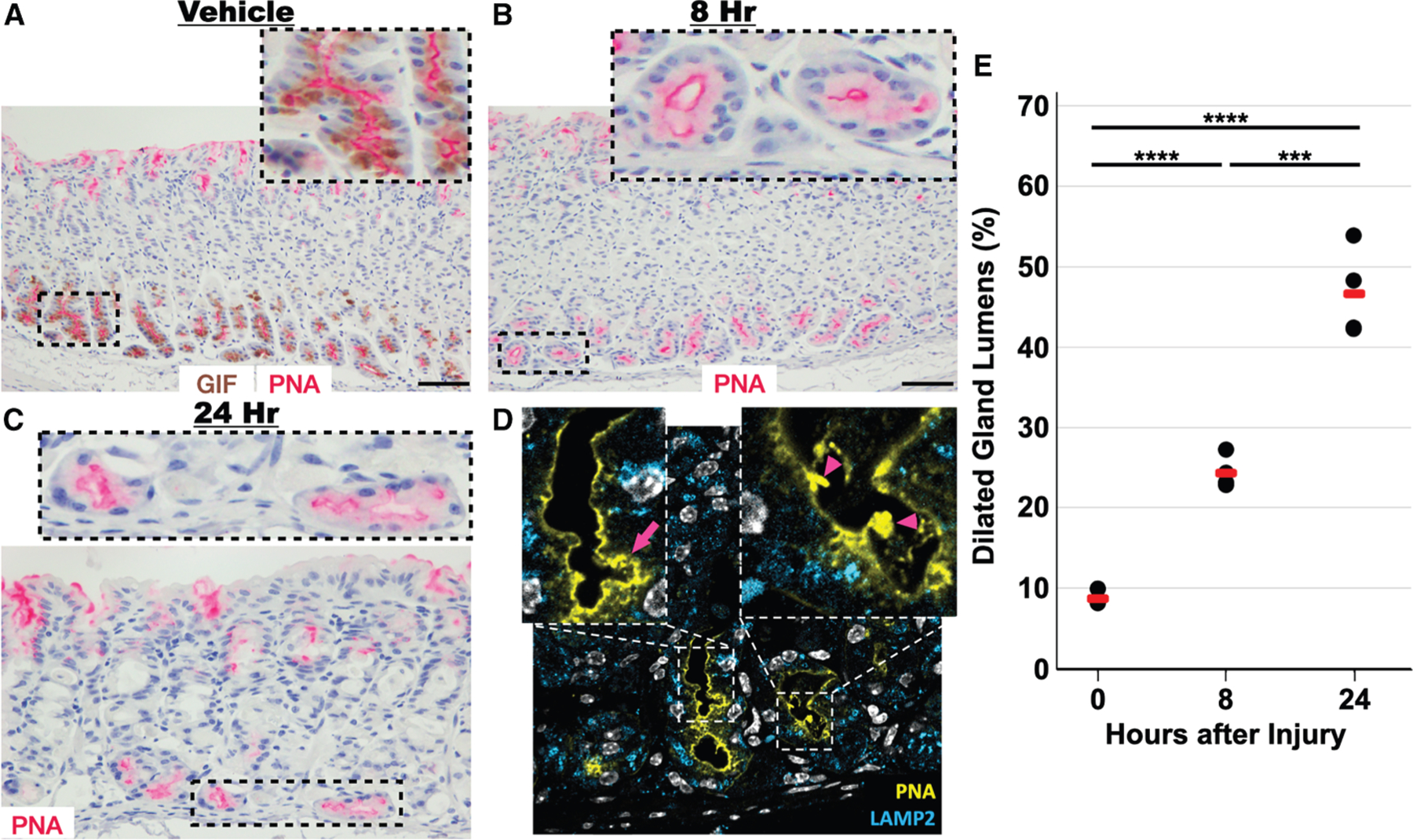
Immunohistochemical and confocal immunofluorescence highlight apical membrane distortion in stage 1 of paligenosis (A) PNA lectin (magenta) is specifically reactive to the apical membrane of chief cells, which can be identified by anti-GIF antibody (brown). (B) 8 h after injury, the gland lumens begin to dilate. (C) At 24 h, the apical membrane of the gastric chief cell becomes convoluted. (D) Using optical sectioning, these convolutions are due to invaginations (magenta arrows) and flaps of membrane (magenta arrowheads), as seen ultrastructurally in Figure 2C. Pseudocoloring: white, nucleus (DAPI); yellow, PNA lectin; blue: Lamp2. (E) Quantification of gland dilation as a function of time after injury. Black dots: mean fraction per individual mouse from >200 fundic glands per mouse; red line: mean of all mice (*n =* 4) per condition depending on the experiment. Significance was calculated with one-way ANOVA of means with Tukey’s multiple comparison test. Scale bars in (A) and (B): 100 μm. Scale bar in (C): 50 μm.

We chose to analyze the 24 h time point in our FIB-SEM studies of the chief cell three-dimensional architecture because (1) at this point, the cells are completing their massive downscaling^4,5^ and (2) because that is when we observed both residual intracellular and newly arising extracellular glycoproteins and ER contents (Figures 1C, 1G, S4C, and S4G). As expected from our earlier work on paligenosis, the FIB-SEM serial sections showed that downscaling cytoplasm harbored various stages of autophagosomes and lysosomal structures; we also noted that the rER had already shrunk considerably from the homeostatic pattern (Figures 2A and 2B). Residual secretory granules were still present (Figure 2B). Note that during downscaling, the cells adopt a more cuboidal cellular shape with retraction of the apical membrane toward the cell base. As the apical membrane retracts basally, the glandular lumen opens, correlating with the expansion of the luminal apical plasma membrane (Figures 3C–3E)^5^; however, we also noted previously uncharacterized, extensive irregularities in the apical cell membranes, including invaginations harboring vesicular-membranous material of different sizes (Figure 2C). The lumens of such vesicular structures were electron-lucent, similar to the extracellular lumen of the gland. Both the electron-lucency and highly variable sizes of these excreted membranous structures would be atypical for exosomes released by the fusion of multivesicular bodies with the apical membrane.

Segmentation (i.e., three-dimensional reconstruction) of the cell beginning at the slice depicted in Figure 2C, using either a concavity (Figures 2D and 2E; Video S1) or convexity (Figure 2F; Video S2) perspective, showed that the electronlucent regions in the apical cell cytoplasm were not enclosed within the cytoplasm but rather topologically part of a network of chambers, all of which ultimately opened to the gland lumen. The process was apical specific, with no detectable irregularities noted in the basolateral plasma membrane.

### Light and confocal microscopy corroborate FIB-SEM ultrastructure

We next studied the apical invaginations at light-microscopic resolution using the lectin peanut agglutinin (PNA), which specifically highlights the chief cell apical plasma membrane (Figure 3A).^31–33^ PNA highlighted the statistically significant, dramatic downscaling of chief cells and widening of the gland lumen beginning at 8 h after high-dose tamoxifen (Figures 3B–3E). At 24 h, cell downscaling was present throughout the gland base, and we could visualize the same convoluted apical plasma membrane with invaginations and membrane flaps seen on FIB-SEM (Figures 3C and 3D). As cells completed downscaling and began to express markers of transition to SPEM (stage 2 paligenosis; 48–72 h after injury) and subsequently reentered the cell cycle, they no longer manifested these contorted apical membranes, although the secreted membranes and glycoproteins (present in the cast-like structures described above) persisted within the lumens of the glands (Figures 1C, 1D, 1G, 1H, and S4). The secreted luminal substance was also visible at 24 h via FIB-SEM (Figure 2B) and in confocal microscopy (Figure S5H).^2,34–36^ Taking these results together, we observed an excretory process of material from multiple cellular compartments (at least ER and zymogenic granules) that occurred during cellular downscaling. The material appeared in the lumen associated with dramatic, long-lived distortion of the apical membrane into a series of invaginations and interconnecting cavities, which does not occur during homeostatic merocrine secretion.

### The excretory process is distinct from autophagy

**Figure 4.**
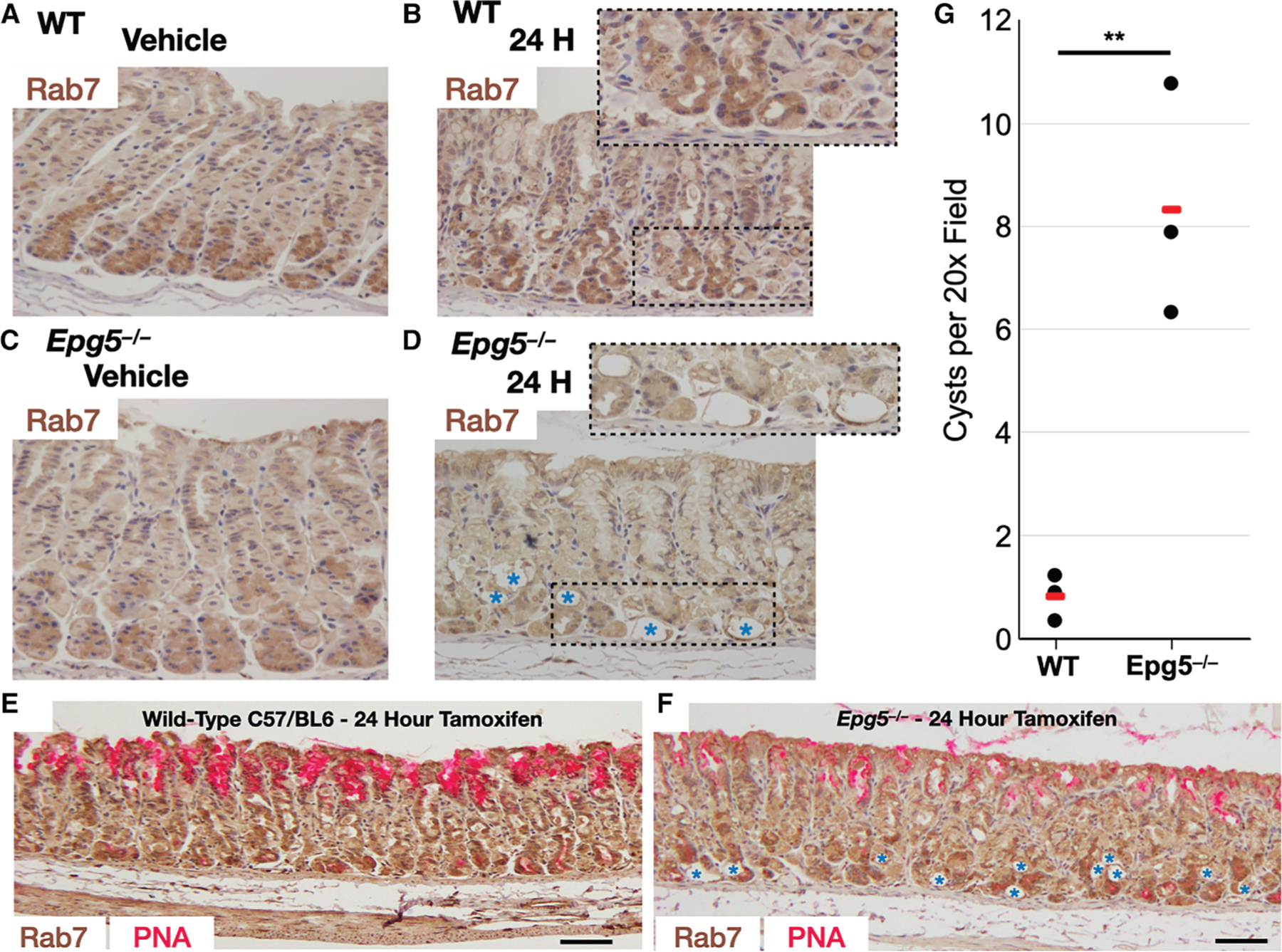
In *Epg5*^−/−^ mice, the late endosome/lysosome fuses with the apical membrane during the cellular downscaling reaction (A and B) Immunohistochemistry (hematoxylin counterstain) with anti-RAB7 of wild-type C57/Bl6 mouse gastric corpus following (A) vehicle treatment or (B) 24 h after injury. (C and D) Immunohistochemistry of age-matched *Epg5*^−/−^ gastric corpus following (C) vehicle treatment or (D) 24 h after injury. (E and F) Wide-field view 24 h after injury, with brown = anti-RAB7 and magenta = PNA (E: WT, F: *Epg5*^−/−^), demonstrating how tamoxifen injury causes cyst formation in the chief cell zones of *Epg5*^−/−^ mice that are mostly absent in wild-type mice. (G) Quantification of cystic structures per high power field 24 h after injury. Black dots: mean fraction per individual mouse from >200 fundic glands per mouse; red line: mean of all mice (*n =* 3) per condition depending on experiment. Significance was calculated by t test. Scale bars: 100 μm.

We next investigated whether lysosomes were involved in the excretory process at the apical membrane. We tracked late LE/Ly structures by immunostaining for the small GTPase RAB7 and the intrinsic membrane glycoprotein LAMP2. At homeostasis, RAB7 marked infrequent, small punctae distributed throughout the cytoplasm (Figures S6A and 4A). After injury, we observed large (up to 5 μm) intracellular RAB7-positive vesicles by 24 h, many of which sequestered cellular sulfated glycoproteins (Figures S6C, S6D, and 5A) (these are difficult to identify in IHC due to the thickness of a 5–7 μm tissue section and a lack of spatial resolution in the enzyme-based IHC assay [e.g., Figure 4B]).

**Figure 5.**
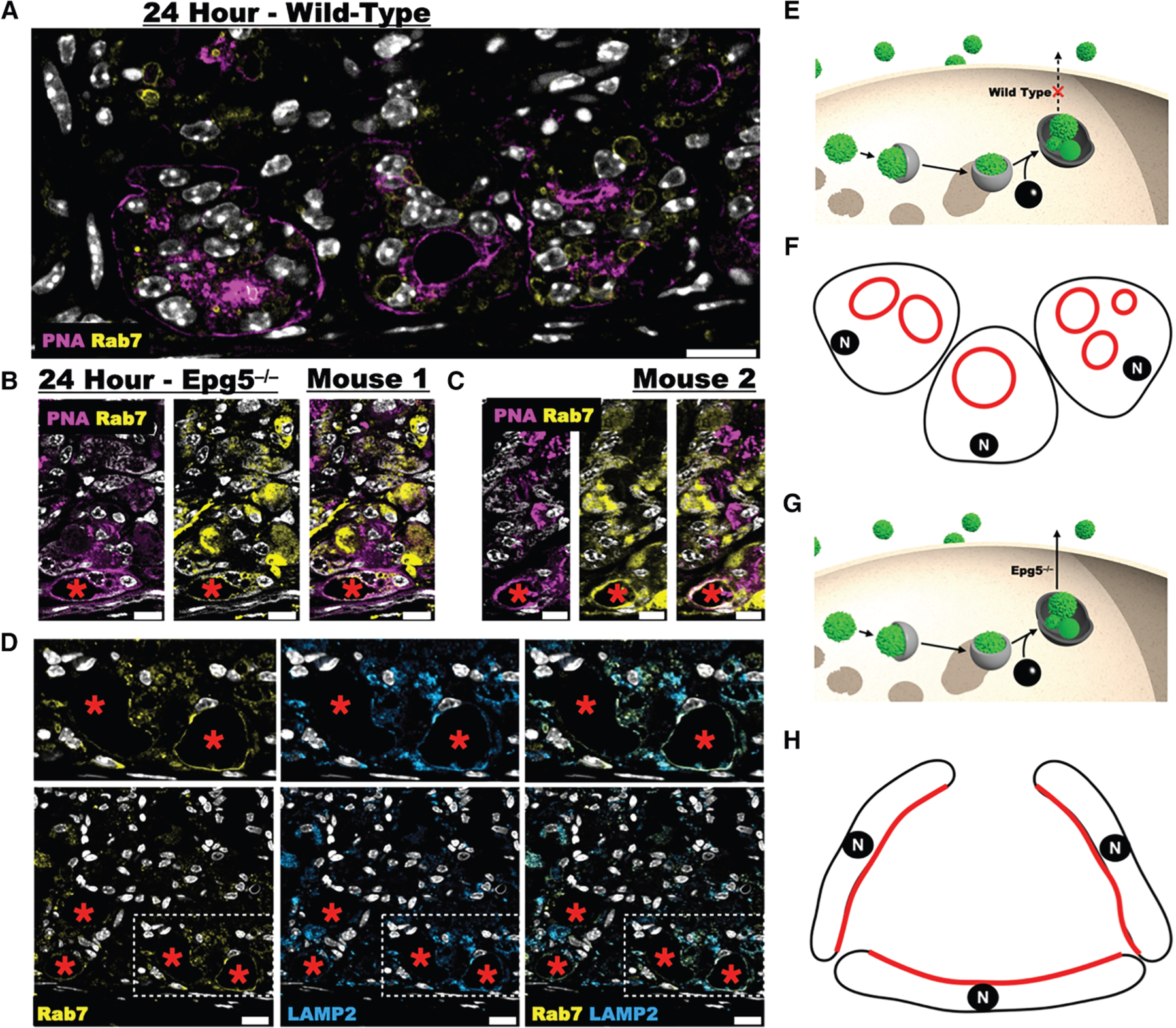
RAB7 does not decorate the apical membrane in wild-type mice but does in *Epg5*^−/−^ mice (A) At 24 h after injury, RAB7 (yellow) is restricted to vesicular structures but does not decorate the apical membrane (magenta, PNA lectin). (B and C) In contrast, in the *Epg5*^−/−^ background, at 24 h, Rab 7 colocalizes with PNA in the cystic structures. (D) Further, the intrinsic membrane protein LAMP2 colocalizes with Rab7 in the apices of the cystic *Epg5*^−/−^ cells. The same apical distribution with immunohistochemistry as in Figure S8. (E and G) Schematic comparing the intracellular trajectory of late endosome/lysosomes in (E) wild-type and (G) *Epg5*^−/−^ mice. (F and H) Excessive fusion of lysosomal/autophagic membranes with the plasma membrane without subsequent retrieval causes such an expansion of apical membrane that cells expand their luminal surface and flatten into cystic structures. N, nucleus. Black: membrane; red: late endosome, lysosome membrane; red asterisks: cyst-like structures. Scale bars: 20 μm.

**Figure 6.**
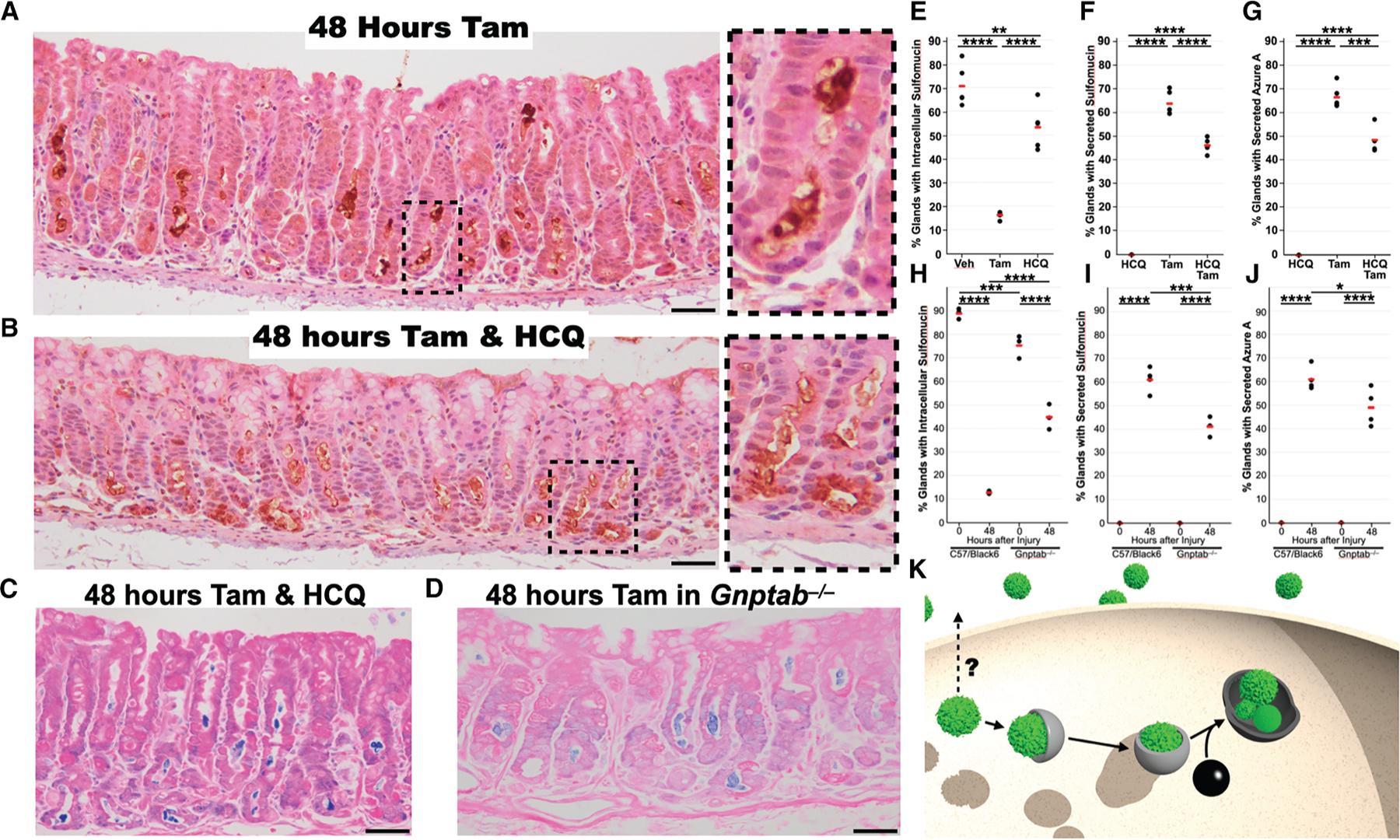
Sulfated mucin excretion requires functioning lysosomes (A) 48 h after high-dose tamoxifen, the chief cells are normally largely devoid of Das-1-positive sulfated mucins (brown; hematoxylin counterstain, blue), with these mucins being found in the extracellular gland lumen. (B) Concurrent administration of hydroxychloroquine with tamoxifen results in intracellular retention of a portion of the sulfated mucins at 48 h post-injury, a time when sulfated mucins are normally absent from the intracellular space. (C) Azure A (blue; eosin counterstain, pink) reactive material is still secreted after concurrent administration of tamoxifen and hydroxychloroquine. (D) Secretion of azure A (blue; eosin counterstain, pink) reactive material in the gland lumen of *Gnptab*^−/−^ mice, which do not have functional lysosomes. Scale bars: 50 μm. Wild-type controls for (C) and (D) are available in Figure 1H. (E) Quantification of the percentage of glands with chief cells harboring intracellular sulfated mucins for homeostatic mice treated with hydroxychloroquine alone, paligenotic glands treated with tamoxifen, or paligenotic glands treated with tamoxifen and hydroxychloroquine. (F and G) Quantification of gland lumens containing secreted sulfomucins or azure A avid material for hydroxychloroquine alone, tamoxifen, or hydroxychloroquine and tamoxifen. (H) Quantification of the percentage of glands with chief cells harboring intracellular sulfated mucins for wild-type or *Gnptab*^−/−^ mice after vehicle or 48 h after injury. (I and J) Quantification of gland lumens containing secreted sulfomucins or azure A avid material for wild-type or *Gnptab*^−/−^ mice after vehicle or 48 h after injury. Black dots: mean fraction per individual mouse from >200 fundic glands per mouse; red line: mean of all mice (*n =* 3–5) per condition depending on the experiment. Significance was calculated with one-way ANOVA of means with Tukey’s multiple comparison test. (K) Schematic representation depicting secretion is mechanistically independent of canonical autophagy that occurs following injury. Gray: classical autophagy; black: lysosome; green: generic cytoplasmic organelle (granule, ER, mitochondria, etc.).

In other cell types, large autophagic vesicles have been described as secreting their contents via direct fusion with the apical membrane (secretory autophagy^37^). The defining component of secretion via this pathway is evidence of intrinsic lysosomal markers rerouted to the apical membrane. Despite extensively searching for the colocalization of lysosomal markers with the apical membrane during paligenosis, we have never observed examples of this pattern in wild-type mice (see, e.g., Figure 5A), suggesting that secretory autophagy is not normally present in chief cells at homeostasis or during paligenosis. The absence of colocalization between lysosomal and apical membrane markers does not absolutely exclude secretory autophagy as a mechanism for extruding the cellular material since the lysosomal proteins could be actively and rapidly reinternalized after secretion. However, given how stable the multi-chambered, apical structures appeared to be, it did not seem likely that there was dynamic flux into and out of the apical membrane during paligenosis. Nevertheless, to determine the role of autophagy in the excretory process occurring in downscaling gastric chief cells, we studied paligenosis in the absence of *Epg5* (ectopic P granules 5 autophagy tethering factor).^38^ EPG5 is a multisubunit tethering complex (MTC) required for the fusion of LC3+ vesicles (autophagosomes) with the RAB7+ LE/Ly.^39^ In contrast to wild-type mice, we observed that RAB7 and LAMP2 localized to the apical membrane in the *Epg5*^−/−^ mice during paligenotic downscaling (Figures 4B vs. 4D, 4E vs. 4F, and 5A vs. 5B and 5C). As would be expected, in wild-type chief cells, LAMP2 colocalized only with intracellular vesicles and often with RAB7 (Figure S7). However, LAMP2 was on the apical membrane in paligenotic *Epg5*^−/−^ chief cells (Figures 4A–4D, 5D, and 6A–6D).

In many *Epg5*^−/−^ paligenotic cells, the extent of incorporation of the LE/Ly membrane into the apical plasma membrane caused the cells to expand their apical membrane, such that the gland lumens became cystic (Figures 4F and 5D, quantified in Figure 4G). EPG5 is a RAB7 effector protein and would be expected to mediate RAB7 localization, so, theoretically, the loss of EPG5 could lead to RAB7—which is membrane associated and not an intrinsic membrane protein—mislocalization to the plasma membrane. However, LAMP2 is an intrinsic membrane protein in LE/Ly, so its presence in the apical plasma membrane is most likely due to the fusion of LE/Ly with the apical membrane and the failure of subsequent intracellular retrieval (Figure 5B).

To summarize the lysosome and plasma membrane trafficking results, we could detect abundant LAMP2 and RAB7 localization to the apical membrane in *Epg5*^−/−^ mice after injury (Figures 4A–4F, 5, and S8), but we could not detect this localization in wild-type mice, even though we observed numerous distortions of the apical plasma membrane associated with the extrusion process at the same time points (see Figure 4G for quantified, summed results). Moreover, the cystic structures that arose from the aberrant fusion of the LE/Ly with the apical membrane in the *Epg5*^−/−^ background were morphologically distinct from the apical structures in wild-type paligenotic cells, as they did not exhibit the elaborate interconnected chambers that invaginate from the apical membrane in wild-type cells. Thus, we conclude that (1) secretory autophagy is unlikely to be the principal mechanism responsible for the extrusion of membranous materials we observe in wild-type mice (Figures 5E–5H) and (2) the wild-type, paligenotic apical invagination network is likely a specific cellular structure (e.g., one that would be supported by dynamic cytoskeletal regulation) because the lower energy state is a flat membrane, as observed in the *Epg5*^−/−^ background.

### Hydroxychloroquine does not block excretion of sulfomucins

We investigated how inhibition of lysosomal function would impact the expression and extrusion of proteins with sulfated mucins. We blocked lysosomal function pharmacologically with hydroxychloroquine, which inhibits vesicular acidification, rendering the lysosomal degradation proteins non-functional, and genetically using mice null for *Gnptab* (N-acetylglucosamine-1-phosphate transferase subunits alpha and beta), encoding a protein essential for trafficking the lysosomal hydrolases to the lysosome.

Treatment with hydroxychloroquine alone significantly reduced the size and apparent number of Das-1-labeled glands at homeostasis (Figures S9 and 6E). During high-dose tamoxifen-induced paligenotic injury, hydroxychloroquine treatment resulted in significantly increased intracellular retention of the Das-1 epitope (Figures 6B–6E), and luminal sulfomucins were slightly reduced but still much more common than in homeostatic glands. Thus, lysosomal inhibition did not completely block cathartocytosis (Figure 6B, quantitated in Figures 6F and 6G). The intracellular persistence of the Das-1 signal could be due to the decreased cathartocytosis of Das-1, but it also could be because hydroxychloroquine blocks whatever fraction of sulfated glycoproteins would normally be degraded by autophagy and lysosomes. Consistent with the conclusion that cathartocytosis does not require autophagy, we observed the same apical membrane deformities even in hydroxychloroquine-treated mice following paligenotic injury (Figure S10).

### FIB-SEM demonstrates that the apical invaginations have phagophore-like activity

**Figure 7.**
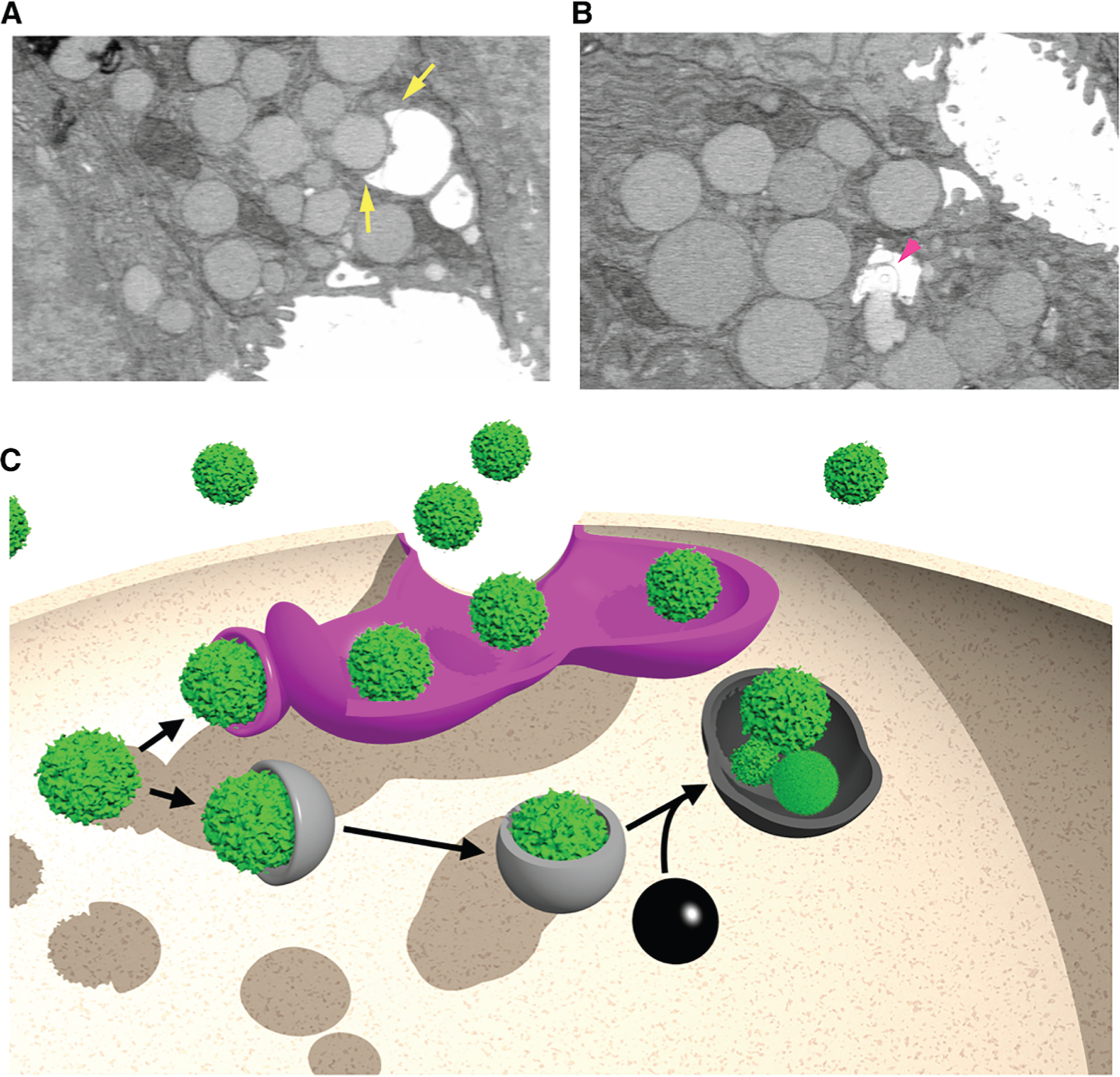
Cathartocytosis model (A) Single two-dimensional sections from FIB-SEM of chief cell apical plasma membrane at 24 h post-injury demonstrate phagophore-shaped structures (i.e., similar to emerging, double-membrane autophagosomes) contiguous with the apical invaginations (yellow arrows). Video S3 shows this section in three-dimensional context. (B) Fusion of a zymogenic granule into an apical invagination with release of cargo as well as membrane into the lumen (pink arrowhead). Video S4 demonstrates the section in three-dimensional context. (C) Schematic representation of canonical autophagy versus our proposed cathartocytosis process. The two processes occur concurrently after injury, and both likely combine to play a role in cellular downscaling. Magenta: cathartocytosis; gray: classical autophagy; black: lysosome; green: generic cytoplasmic organelle (granule, ER, mitochondria, etc.).

In further characterizing the apical membrane deformations occurring during stage 1 of paligenosis, we observed phagophore (cup)-shaped structures emanating from the apical invaginations (Figure 7A; Video S3). Additionally, we observed the direct fusion of a zymogenic granule with the apical invaginations. Although the fusion of cellular organelles with the invaginated apical network resulted in outward diffusion of vesicular contents (smooth gradient of electron density in Figure 7B), it also generated extracellular membranous material (arrowhead in Figure 7B), which is not characteristic of simple merocrine secretion (Figure 7B; Video S4). Although the membrane released into the lumen of the apical invaginations appears to originate at sites of organellar docking and release, we are unable to determine if the released membrane derives from the apical invagination or docked vesicle.

## DISCUSSION

Using a synchronous *in vivo* murine model of gastric injury-induced metaplasia, we describe how mature, differentiated cells downscale their cellular machinery en route to a proliferative phenotype. Our work supports the importance of lysosomes and autophagic machinery in cellular downscaling (Figure 7), as has previously been reported.^2,4,5^ However, we also uncover a cellular process that occurs concurrently with, but does not utilize, autophagic machinery. We propose the term cathartocytosis (in Greek, cellular cleansing) for this cellular process, which bestows a previously undescribed excretory capacity of cellular structures through the apical membrane.

Cellular plasticity is paramount for the resurrection of the cell census and maintenance of barrier function following substantial tissue injury.^40,41^ Cellular reprogramming to a regenerative state can supersede the homeostatic functions of differentiated cells when the injury is severe enough to potentially compromise the barrier. This is especially true for the gastrointestinal epithelium, which plays an exceedingly important barrier function, protecting the human body against ingested and endogenous caustic, toxic, and pathogenic material to which it is constantly exposed.

Our group has previously described a stereotyped series of cellular-molecular events that occur in differentiated cells following injury to license these cells to proliferate. This process, called paligenosis,^4^ results in differentiated cells downscaling their cellular machinery to reenter the cell cycle. Here, we studied how these dramatic phenotypes/architectures come about.

An essential early step in paligenosis is the upregulation of the transcription factor *Atf3*, which, in turn, induces expression of *Rab7*.^5^ Here, we demonstrate that canonical autophagy is responsible for the removal of some but not all of the intracellular mature differentiated features and that the cell’s autodegradative machinery is supported by the direct excretion of cellular organelles through the apical membrane to help achieve rapid cellular downscaling (Figure 7C). Further, our analysis indicates that such extrusion is an active secretory process as opposed to the simple sloughing off of glandular material membranous material, as was previously proposed.^36^

The cytoplasmic compartment of the gastric chief cells is mostly occupied by an expansive rER and zymogenic granules localized apically to the nucleus (Figures 2A and S5). While, as we previously have reported, some of the ER and granules are indeed degraded by lysosome-dependent machinery, we were also able to show that another portion of these organelles were jettisoned into the apical lumen by a process we term cathartocytosis. We do not believe that what we observe is exosome secretion since exosomes are characterized as a relatively uniform population of vesicles secreted by the fusion of the multivesicular body with the apical membrane.^42^ Further, by transmission electron microscopy, exosomes are approximately as electron dense as the cytosol because they are formed intracellularly and secreted following the fusion of the multivesicular body with the apical membrane. In contrast, the excreted vesicular lumens we observe are electron-lucent. In the case where we observed a secretory granule interfacing with the apical plasma membrane, there was a gradient of decreasing electron density as the secreted substance neared the gland lumen (Figures 7B; Video S4). Lastly, exosomes are formed from the invagination and sequestration of cytoplasmic material into the multivesicular body, which then fuses with the apical membrane. As the glycosylation epitopes on mucins are never exposed to the cytosol, the only way for mucins to end up as cargo in the much smaller exosomes would be for the very large (1 μM) secretory mucin vesicles to be enveloped by the multivesicular body, which would then fuse with the apical membrane. However, we observe neither obvious multivesicular bodies in the cytoplasm at any time point nor the excretion of double membranes (exosome and secretory vesicle inside) that we would expect from this mechanism (Figure 7). We speculate that the extracellular membranous material we observe being extruded is variegated and frequently vesicle-like because the membranes (1) were partially lysed during their generation, (2) have fenestrations, (3) formed secondarily in the extracellular milieu from jettisoned lipid material, and/or (4) formed around extraluminal material. Based on the ultrastructural characterization here, we favor one of the first two mechanisms (Figure 7B; Video S4).

In the cases where we observed transitional structures, the secreted material appeared to be released following docking, with phagophore-shaped structures arising from the apical membrane invaginations independent of a multivesicular body (Figures 7A and 7B; Video S4; see also graphical abstract). The secreted vesicular material we observe is distinct from oncosomes, microvesicles, ectosomes, apoptotic bodies, and exophers, as the membrane topology of all these vesicular structures involves budding from the apical membrane.^42^ Other secretory mechanisms have been described involving the fusion of organelles containing other vesicles, like secretory lysosomes^37,43^; however, we have ruled out a significant contribution from secretory lysosomes in wild-type mice using the *Epg5* null mice as positive controls to show that we have the tools to detect this process yet do not observe it in wild-type paligenotic cells. Further, we are not aware of another secretory mechanism that involves the formation of an expansive, elaborate, long-lived, ant-hill-like network of the apical membrane. Lastly, we observe cathartocytosis to occur as a means of rapidly downscaling cellular contents and size, while other secretory processes roughly maintain cell size and contents.

During times of profound exocytosis, which occurs in processes like merocrine secretion, limiting the excessive incorporation of the membrane to the apices of the cell is of paramount importance to maintaining homeostatic cell shape and size. For example, in *Drosophila*, during exocrine secretion from the larval salivary gland, it was found that following vesicle fusion, the remanent membrane is not incorporated into the apical membrane but instead is supported by the actomyosin cytoskeleton and subsequently crumples and is removed by endocytosis.^44^ Here, we describe an elaborate set of invaginations from the apical membrane that are likely also supported by the cytoskeleton. However, the apical invaginations described in this manuscript are distinct from vesicle crumpling described in *Drosophila* because (1) the size is much larger than a single secretory vesicle and the complex, cavernous network is much more elaborate than vesicle crumpling; (2) vesicle crumpling was shown to be a means of eliminating excess membrane from the apices by endocytosis, while the membrane invaginations in cathartocytosis serve as an excretory apparatus; and (3) the apparatus we describe is stable, lasting for hours, while the vesicle crumpling resolves within minutes. It is unclear at this time where the membrane used to create these large stable apical invaginations derives from.

The excretory mechanism of cathartocytosis is distinct from merocrine secretion for a number of other reasons. The elaborate, stable apical invagination network that develops to jettison the material is not a feature of merocrine secretion. Further, the magnitude of material released from the cell (in conjunction with autophagy) results in a significant reduction in cell size (Figure S3). This is in contrast to merocrine secretion, which preserves cell size. Lastly, we hypothesize that the function of cathartocytosis is different from merocrine secretion. In merocrine secretion, the cell secretes a particular substance (digestive enzymes, protective mucus, neurotransmitters, etc.) that serves a specific useful purpose for the organism, while the material excreted with cathartocytosis is more heterogeneous and aids in downscaling during a cellular transformation. Although zymogenic cells like chief cells are not known to undergo apocrine secretion, cathartocytosis is distinct from this process in that apocrine secretion results from budding/fragmenting of the apical cytoplasm, while cathartocytosis involves fusion events with an invaginated apical network.

At a cellular level, the function of EPG-5 was originally identified in a *C. elegans* autophagy screen, where it was suggested that it played a role during the late stages of autophagocytic degradation.^45^ Consistent with this role, it was later shown in cell lines that *EPG5* functions as a RAB7 effector, augmenting soluble NSF attachment protein receptor (SNARE)-mediated fusion of the late endosome with the LC3+ autophagophore.^39^ In the absence of EPG5, these authors found that RAB7 vesicles were promiscuous and fused with other compartments, including early endosomes. Here, we found that following injury in the absence of EPG5, the RAB7+/LAMP2+ LE/Ly fused with the apical membrane, an outcome we have not seen before in paligenosis in any other context (Figures 5, 6, S6, and S7). Moreover, the *Epg5* null paligenotic cells demonstrate a far more dramatic reduction in apical-basal distance/thinning out of the cells, likely a consequence of (1) the addition of LE/Ly membranes to the apical domain and (2) cell death, resulting in the stretching of the remaining viable cells. Fusion of the LE/Ly with the apical membrane occurs at homeostasis in the osteoclast and is important for bone resorption and is described as secretory lysosomes.^37^ Thus, it appears that EPG5 plays an important role in limiting excessive incorporation of the membrane into the apices of the cell, presumably by limiting inappropriate SNARE complexes from forming. It remains to be determined whether such aberrant trafficking of the LE/Ly in *Epg5* null cells is responsible for the known immunodeficiency in humans with Vici syndrome, which is characterized by mutations in *EPG5*. The dramatic phenotypic difference between injured wild-type cells, which are cuboidal with stable apical invaginations, and injured *EPG5*^−/−^ cells, which are pancake-shaped, demonstrates the importance of (1) regulating which membrane compartments are incorporated into the extracellular membrane and (2) cytoskeletal support of secretory structures/invaginations.

The reason why hydroxychloroquine results in smaller zymogenic secretory granules is not clear at this time (Figure S9). There is minimal lysosomal activity in the uninjured stomach, and there is no evidence that secretory vesicles derive from lysosomes. Hydroxychloroquine has also been described to affect Golgi trafficking and other endosomal processes, which seems more likely to be responsible for the hydroxychloroquine zymogenic granule phenotype.^46,47^ Indeed, we have previously found that a mannose-6-phosphate receptor-domain-containing protein, ELAPOR1 (endosome/lysosome-associated apoptosis and autophagy regulator 1), is also involved in chief cell granule maturation.^48^ As might be discerned from its name, ELAPOR1 is known to regulate both endosome and lysosome trafficking. We found that hydroxychloroquine treatment slightly reduced, but did not block, the secretion of sulfomucins (Figure 6), nor did it prevent the formation of apical membrane deformities characteristic of cathartocytosis (Figure S10). We did observe that hydroxychloroquine promotes the retention of sulfomucins within the cell (Figure 6E), which we speculate is largely due to an inability of the lysosome to digest the sulfomucins allocated to it during injury.

The fact that sulfated glycoproteins (likely mucins; Figure S2) dynamically redistribute during metaplasia may explain why sulfated mucin expression is heterogeneous/variegated when viewed in chronic metaplasia in humans.^20^ Such metaplasia would be subject to chronic inflammation and repair in a non-synchronous fashion so that we might observe cells at various states of sulfated mucin trafficking, as has been reported by us and others in Barrett’s esophagus,^24^ intestinal metaplasia of the stomach,^24,49^ and pancreatic intraepithelial neoplasia 3.^19,20^

We show here that murine zymogenic granules contain 3ʹ-sulfo-Le^A/C^ mucins (Figure 1) at homeostasis. They also harbor damage-associated molecular patterns (DAMPs) like cathelidicins (LL-37/hCAP8). Both sulfated mucins and cathelidicins bind the pathogenic organism that causes chronic metaplasia and increases the risk for gastric cancer in hundreds of millions of people worldwide: *H. pylori*.^50–52^ The binding has been best worked out for sulfated mucins, where they associate with neutrophil-activating protein (HP-NapA) in a pH-dependent fashion.^51,53,54^ The importance of HP-NapA as a virulence factor is illustrated by the protective effect of vaccination with HP-NapA prior to *H. pylori* challenge.^55^ Thus, cathartocytosis may serve not just as a means of helping cells downscale rapidly in paligenosis but as a way to help bind and flush *H. pylori* out of the gland lumen. Other defensins are overexpressed and secreted in response to *H. pylori* and may also serve similar functions.^56–59^

In addition to binding *H. pylori*, the 3ʹ-sulfo-Le^A/C^-laden mucins also bind several host immune receptors.^20^ For example, 3ʹ-sulfo-Le^A^ is the most potent ligand for E- and L-selectins.^60–65^ The cysteine-rich domains of the macrophage mannose receptor as well as the dendritic cell immunoreceptor also have affinity toward 3ʹ-sulfo-Le^A/C^.^66,67^ Thus, the secretion of sulfated mucins following injury that causes metaplasia or occurs in the cancer microenvironment could alter the immune response.

Overall, here we describe the membrane-shuttling trajectories used by injured cells to downscale their cellular machinery en route to the metaplastic phenotype. Our data reinforce that canonical autophagy serves an important role but also uncover a previously undescribed cellular process that we call cathartocytosis and that bestows excretory capacity to the apical membrane, which may help cells not only downscale to a smaller, progenitor-like state during paligenosis and regeneration but may also serve as an anti-microbial mechanism or immunomodulatory function. In the future, we hope to explore if cathartocytosis is common to, and restricted to, paligenosis (which is broadly conserved across organs and species) and/or if it also occurs in injured cells that are not undergoing massive activation of cell plasticity.

### Limitations of the study

This study is limited in that we do not yet know the cellular and molecular mechanisms underpinning cathartocytosis. For example, as discussed above, cellular downscaling is decreased by the blockade of lysosomal function, but cathartocytosis, (sulfomucin extrusion) from cells is decreased but not abrogated. This suggests that lysosomes are not needed for cathartocytosis; however, there are other interpretations. The current study is also limited to cathartocytosis during paligenosis in gastric chief cells. We do not yet know if cathartocytosis is universal to paligenosis. It seems highly unlikely that this cellular process, however, would have evolved for only a single cell type. But we are limited in our models to study paligenosis, with gastric chief cell injury being among the best for its synchronicity and rapidity. A boon for the field would be the development of *in vitro* models. The lack of *in vitro* paligenosis models is likely because the objective of tissue culture, even in organoid models from adult tissue, is to make cells proliferate to propagate them, not to maintain them in a differentiated, physiological state surrounded by appropriate extracellular signals from the matrix and other cell types with circulating blood.^68–76^ Thus, whereas paligenosis is the conversion of such fully differentiated cells into proliferating progenitors, tissue culture is chiefly a means of propagating such progenitors.

## RESOURCE AVAILABILITY

### Lead contact

Further information and request for resources and reagents should be directed to and will be fulfilled by lead contact, Jeffrey W. Brown (brownjw@wustl.edu).

### Materials availability

All mouse lines and materials used in this study were provided or purchased from the mentioned companies or researchers. This study did not generate any new or unique reagents. If the mouse lines are still in the investigator’s colony, we are happy to share them.

### Data and code availability

- Data are available upon request.
- The paper does not report original code.
- Any additional information or data are available from the lead contact upon request.

## STAR★METHODS

### EXPERIMENTAL MODEL AND STUDY PARTICIPANT DETAILS

#### Animals

All experiments using animals followed protocols approved by the Washington University in St. Louis, School of Medicine Institutional Animal Care and Use Committee. WT C57BL/6 mice were purchased from Jackson Laboratories (Bar Harbor, ME). *Epg5*^−/−^ mice were obtained from breeding *Epg5*^*+/*−^ mice, a kind gift from Megan Baldridge.^77–79^ Likewise, *Gnptab*^−/−^ were obtained by breeding *Gnptab*^+/−^ mice (A kind gift from Stuart Kornfeld).

Tamoxifen powder (Toronto Research Chemicals) was initially solubilized in 100% ethanol via sonification after which it was emulsified in sunflower oil (Sigma-Aldrich) at a 9 Oil:1 EtOH ratio.^80^ Tamoxifen (5 mg/20 g body weight; Toronto Research Chemicals) was injected intraperitoneally daily for up to 2 days or until mouse was euthanized for histologic examination. Hydroxychloroquine (120 mg/Kg by intraperitoneal injection) was administered 24 h prior to the first tamoxifen injection and then with all subsequent tamoxifen injections until the tissue was harvested. All mouse experiments were performed on mice aged 6–10 weeks and both sexes were utilized indiscriminately as prior studies have demonstrated an identical phenotype.^25–27^

### METHOD DETAILS

#### Imaging and tissue analysis

Following anesthetizing with isoflurane and cervical dislocation, murine stomachs were excised, flushed with phosphate buffered saline and fixed overnight with 10% formalin. They were washed and equilibrated in 70% ethanol for several hours prior to embedding in 3% agar and routine paraffin processing. Sections (5–7 μM) were prepared for immunohistochemistry and/or immunofluorescence by deparaffinization using Histoclear and an alcohol series for rehydration. After which antigen retrieval was performed in 10 mM citrate buffer, pH 6.0 in a pressure cooker. Tissue was blocked with 2% BSA and 0.05% Triton X-100. Primary and secondary antibodies were diluted in 2% BSA and 0.05% Triton X-100. Vector ABC Elite kit was used for immunohistochemistry. Immunohistochemistry slides were mounted with Permount and immunofluorescence with Prolong Gold. Brightfield images were taken on either a Nanozoomer (Hamamatsu 2.0-HT System) for quantitation or Olympus BX43 light microscope. Confocal images were obtained on a Zeiss LSM880 confocal microscope.

#### Azure A staining

Slides of paraffin embedded tissue were dewaxed and rehydrated with an ethanol series to 30%. Antigen retrieval must not be performed as this results in loss of staining. Slides were then stained with 0.1% Azure A Chloride in 30% ethanol for 1 min. Slides were then washed in water. Placing slides in citric acid/sodium phosphate buffer, pH 4 results in royal blue nuclear staining and dark blue cytoplasmic staining. The slides were then transferred to 95% ethanol and stained with Eosin Y. The slides were subsequently mounted with permount.

#### Focused-ion beam scanning electron microscopy

The tissue fixation and acquisition parameters have been previously described.^5,81^ Three-dimensional reconstructions of either the gastric chief cells or the gland lumen were obtained by initially masking using ilastik-1.4 using the carve protocol.^82^ These masks were exported to FIJI (ImageJ)^83^ to crop and create an image stack. This image stack was then imported to ChimeraX^84^ to generate and render the reconstruction. Movies form the rendered images with iMovie.

#### Western blot

Western blot samples were prepared using ∼30 μg of protein in standard SDS-PAGE lammeli buffer with 5% β-mercaptoethanol. Samples were heated to 70°C for 10 min prior to running through NuPAGE precast gels. Protein was transferred in NuPAGE transfer buffer containing 20% methanol to nitrocellulose which was blocked using 5% BSA in PBS. Nitrocellulose membrane was incubated with primary antibodies in blocking buffer overnight at 4°C. Membranes were washed with PBS and then incubated in appropriate secondary antibodies at 1:10,000 dilution in blocking buffer. After extensively washing, membrane was imaged using LiCOR Odyssey.

#### Dot blot

Sample was applied to nitrocellulose using a BIORAD vacuum apparatus, after which the nitrocellulose was stained and imaged in an identical fashion to western blots.

#### Chloroform extraction

Flowchart is displayed in Figure S2D. 24 h after a C57BL/6 mouse was injected with tamoxifen (See above), the 50 mg of stomach tissues was excise and homogenized in 0.5 mL of phosphate buffered saline w/protease inhibitors. To 0.2 mL of this homogenate, 1.2 mL of methanol and 2 mL of chloroform was added and incubated at 37C in a shaker. An additional 1 mL of methanol was added, and the mixture was spun at 2000g for 30 min at 20C. The majority of the supernatant was removed; however, since the pellet was still soft it was spun at 20,817g for an additional 2 min and the remaining supernatant removed. The pellet was resuspended in 2 mL of 1:2:0.8 (chloroform:methanol:water) and 10% of the solution was banked for dot blot analysis (Pellet #1). The remaining solution was shaken at 37C for 2 h. After which the slurry was spun at 20,817g for 2 min. The supernatant was removed and added to the prior supernatant and the pellet banked for analysis (Pellet #2). The supernatant and both pellets were dried prior to be resuspended in 4% SDS (supernatant and Pellet #1) or 4% SDS and 6M Urea (Pellet #2). Equivalent volume percentages were loaded for dot blot analysis.

#### Deglycosylation

Mucins in solution were treated with O-Glycosidase, Neuraminidase, or PNGaseF according to manufacturer’s/New England Biolab’s instructions.

Slides of paraffin embedded tissue were dewaxed and rehydrated, followed by antigen retrieval as described above. To remove O-linked glycans the sections were incubated in 0.1N NaOH for 2 h at 20C (Longer incubations or higher temperatures result in tissue being dislodged). The slides were then transferred to phosphate buffered saline and assayed as described above. N-linked glycans were removed by treating with PNGaseF for 2 h at 37C. Slides were also treated with O-glycosidase and neuraminidase for 2 h at 37C.

#### Fundic chief cell size

2-Dimensional area measurements of chief cells were made by analyzing 40x micrographs of hematoxylin and eosin stained sections. Cells were outlined manually and area calculated with ImageJ/FIJI.^83^ 270 pixels/50 μm. Only well-defined chief cells within 3 cells of the base of gland with both nuclei and luminal apex present were included in the calculations.

### QUANTIFICATION AND STATISTICAL ANALYSIS

All statistical details can be found in the figure legends.

## Supplementary Material

1

2

3

4

5

Supplemental information can be found online at https://doi.org/10.1016/j.celrep.2025.116070.

## Figures and Tables

**Table:** KEY RESOURCES TABLE

REAGENT or RESOURCE	SOURCE	IDENTIFIER
Antibodies		

Rat Anti-BrdU @ 1:200	Abcam	AB6325
Mouse Anti-Trap-Alpha @ 1:100	Santa Cruz	Sc-373916
Rabbit Anti-Sox9 @ 1:5000	Sigma	AB5535
Biotinylated PNA Lectin @ 1:1000	Vector	B-1075-5
Rabbit Anti-PDI @ 1:500	Cell Signaling	3501
Rabbit Anti-Rab7 @ 1:200	Abcam	Ab137029
Rabbit Anti-LC3B @ 1:100	Novus	NB600-1384
Biotinylated GSII Lectin @ 1:1000	Vector	B-1215-2
Anti-Sulfo-Le^A/C^ (Das-1)	Leinco	Inhouse
Rabbit Anti-GIF (see Figure S10 for Validation of its use in Mice)	Thermofisher	PA5-59293
Rat Anti-Human CD44 @ 1:200	Cosmo Bio	CAC-LKG-M003
Rat Anti-Mouse CD44 @ 1:200	Cosmo Bio	CAC-LKG-M002
Goat Anti-GIF	N/A	Inhouse

Chemicals, peptides, and recombinant proteins		

PNGaseF	New England Biolabs	P0704
O-Glycosidase	New England Biolabs	P0733
Neuraminidase	New England Biolabs	P0722
Permount	Fisher	SP15
Prolong Gold with DAPI	Fisher	P36971
Histoclear	National Diagnostics	HS-200
Azure A	Sigma	A6270
Sunflower Oil	Sigma	8001-21-6
Hydroxychloroquine	Acros Organics	CAS#747-36-4
Tamoxifen	Toronto Research Chemicals	TRC-T006000

Critical commercial assays		

Vectastain Elite ABC-HRP Kit	Vector Laboratories	PK-6100

Experimental models: Organisms/strains		

Global Epg5 Knockout Allele	Megan Baldridge	Bred Het x Het
Global Gnptab Knockout Allele	Stuart Kornfeld	Bred Het x Het
C57BL/6	Jackson Laboratory	

Software and algorithms		

ChimeraX		https://www.cgl.ucsf.edu/chimerax/
Chimera		https://www.cgl.ucsf.edu/chimerax/older_releases.html
FIGI (ImageJ)		https://imagej.net/software/fiji/downloads
iMovie		apple.com
Ilastik v1.4		https://www.ilastik.org/download

Other		

Nanozoomer	Hamamatsu	2.0-HT System
FIB-SEM	Zeiss	Crossbeam 540
Light Microscope	Olympus	BX43
Confocal Microscope	Zeiss	LSM880

## Highlights

- Defining cathartocytosis, an injury-induced process cells use to downscale cellular machinery
- Three-dimensional reconstruction detailing how cells reorganize organelles during paligenosis
- Cathartocytosis and autophagy occur together in paligenosis but are mechanistically distinct
- EPG5 prevents fusion of autophagic compartments with the apical membrane after injury
