# Supplementary material for: Cathartocytosis: Jettisoning of cellular material during reprogramming of differentiated cells
Source: Cell Rep. Author manuscript; Available in PMC 2025 Sep 29. (PMC12478994; doi:10.1016/j.celrep.2025.116070)
Supplement: 1 [file NIHMS2107265-supplement-1.pdf]

Cell Reports, Volume *44*

Supplemental information

Cathartocytosis: Jettisoning of cellular material during reprogramming of differentiated cells

Jeffrey W. Brown, Xiaobo Lin, Gabriel Anthony Nicolazzi, Xuemei Liu, Thanh Nguyen, Megan D. Radyk, Joseph Burclaff, and Jason C. Mills

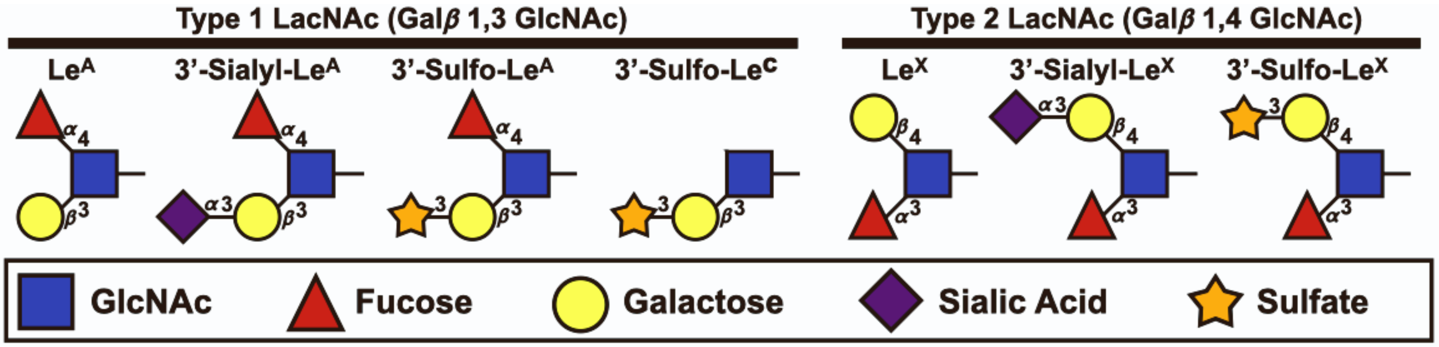

Supplemental Figure 1. Schematic representation of relevant Lewis glycans. Type 1 differs from Type 2 in the arrangement of the galactose and fucose about the N-Acetylglucose. The antibody Das-1 recognizes 3'-Sulfo-Le^{A} and 3-Sulfo-Le^{C}, the difference between the glycans being 3'-Sulfo-Le^{C} lacks a fucose. 3'-Sialyl-Le^{A} is the oncoantigen CA19-9.

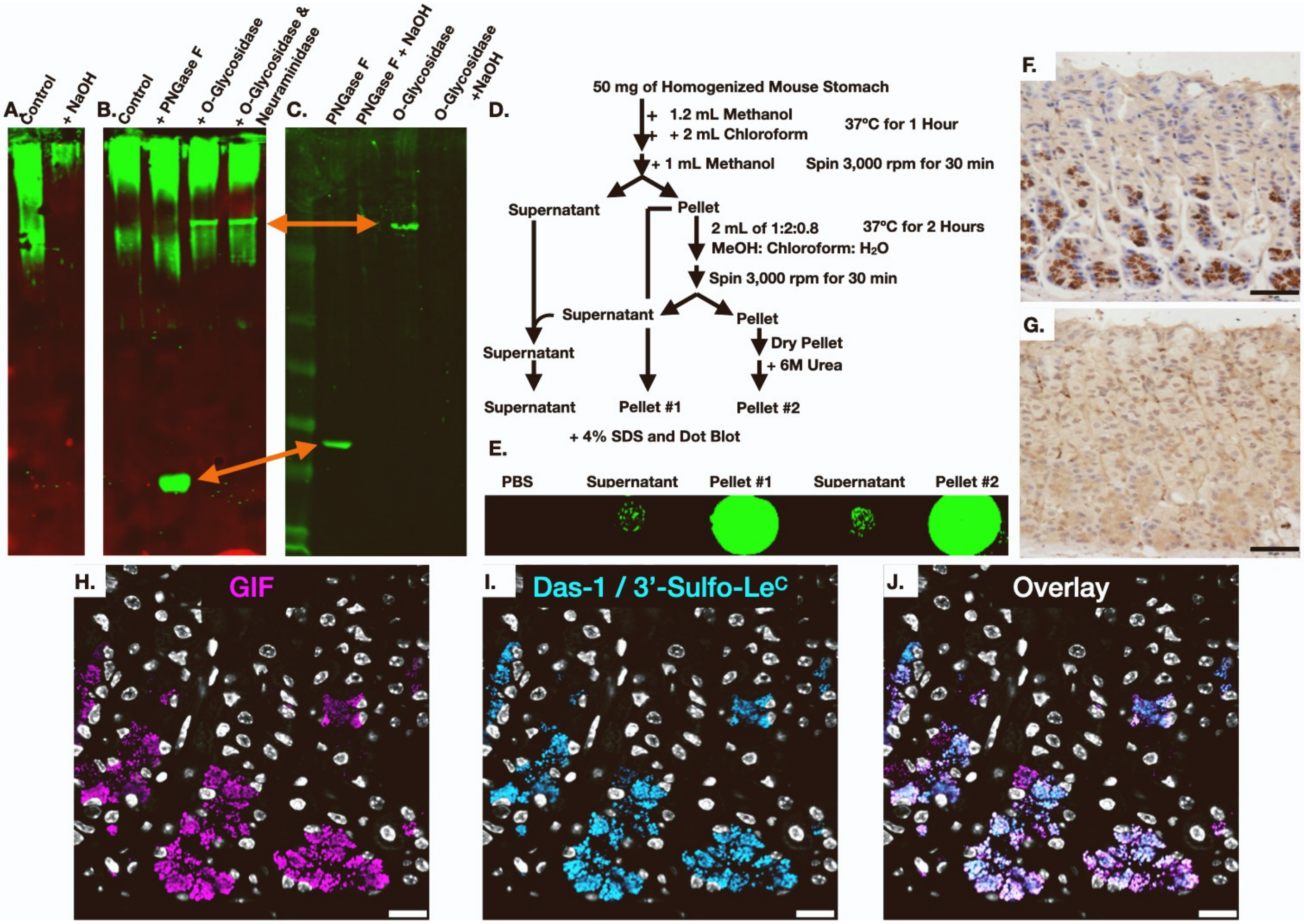

Supplemental Figure 2. 3'-Sulfo-Le$^{A/C}$ is present on large O-linked glycoproteins and present within the apical zymogenic granules. Concentrated conditioned media from containing LS174T cells were used as the standard for 3'-Sulfo-Le$^{A/C}$ substance [1]. **A**. Western blot of 3'-Sulfo-Le$^{A/C}$ standard showing NaOH treatment to strip glycans and sulfates results in loss of most signal. **B**. Western blot of 3'-Sulfo-Le$^{A/C}$ standard following treatment of PNGase F (which releases N-linked glycans) and O-Glycosidase (which releases, simple, unbranched O-glycan) with and without Neuraminidase (which releases sialated glycans). No change in Das-1 intensity was noted in any of the conditions. The resistance of Das-1 epitope to those conditions indicate it is a sulfated glycan on branched O-linked tree; however, note that PNGase likely shifts Das-1-labeled bands to lower molecular mass due to loss of N-linked glycans elsewhere on the protein backbone. **C.** At the high concentrations of recombinant enzymes recommended by the manufacture to use in these deglycosylation assays, we observed Das-1 reactivity on the recombinant enzymes run in lanes by themselves (arrows between Panel B and Panel C). Note both PNGaseF and O-Glycosidase (pure reagent as supplied by NEB) bind

Das-1 and that this reactivity is lost after treatment with NaOH. It is unclear whether this is due to these recombinant proteins expressed in bacteria containing 3’-Sulfo-Le$^{A/C}$ epitopes or non-specific binding with proteins at high concentrations. Again, in any case, the removal of the glycan with sodium hydroxide, but not O-glycosidase is consistent with 3’-Sulfo-Le$^{A/C}$ residing on a branched O-Linked mucin. **D.** Protocol flowchart for chloroform extraction. **E**. Dot blot of PBS alone, first supernatant, first pellet, second supernatant, second pellet. The signal in the aqueous (pelleted) but not chloroform (supernatant) fractions demonstrates 3’-Sulfo-Le$^{A/C}$ is on a glycoprotein and not hydrophobic moiety like a lipid. **F, G**. Immunohistological confirmation that Das-1 reactivity is lost following (**G**) treatment with NaOH relative to control (**F**). Scale bars = 50 µm. H-J. Confocal microscopy of the 8 hour time point, chosen in the unlikely event that injury induced a new population of secretory vesicles in paligenotic cells, demonstrates a similar subcellular location of GIF (purple) and the Das-1 epitope (cyan) in chief cell secretory vesicles. Scale bars = 20 µm.

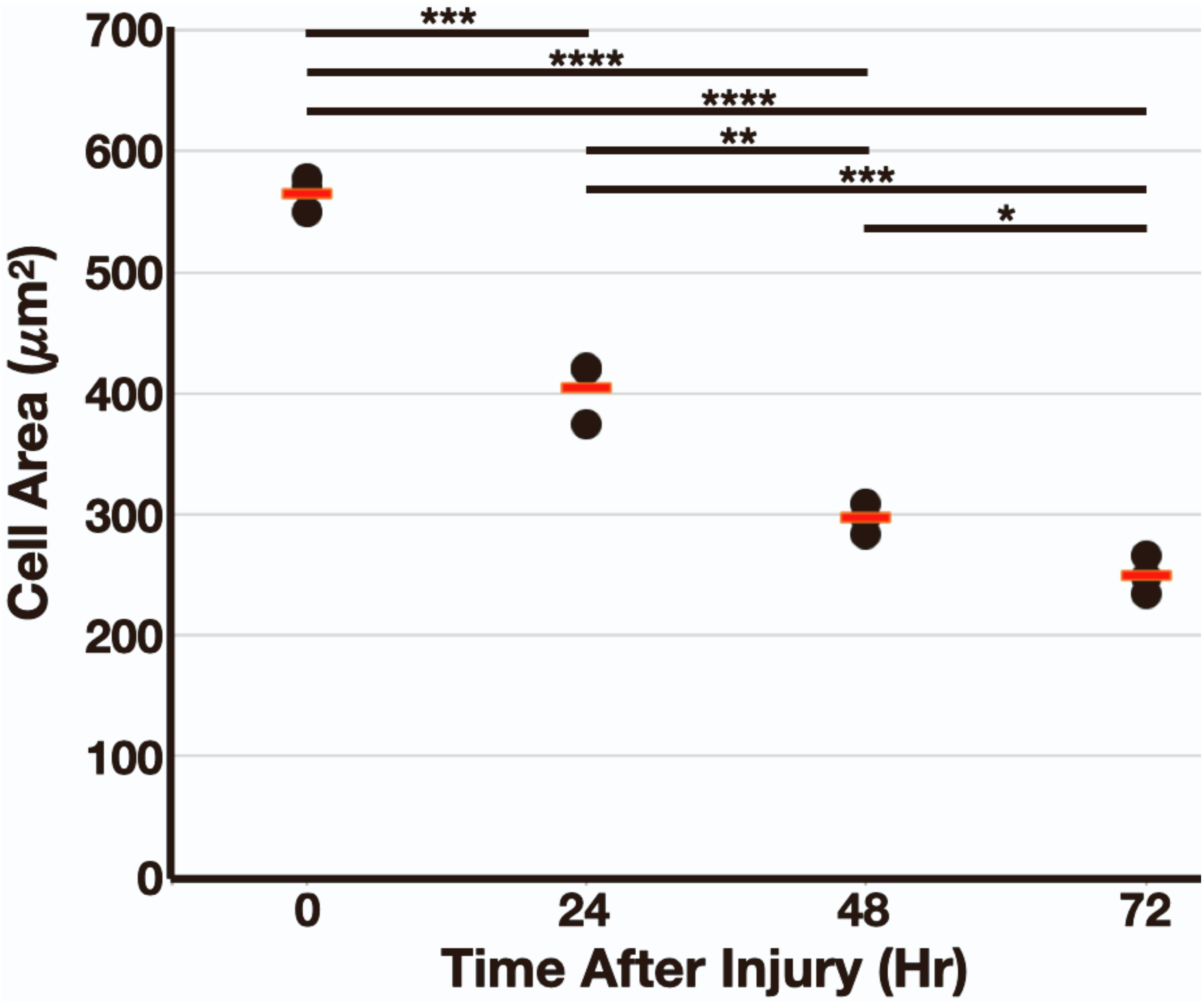

Supplemental Figure 3. Quantification of chief cell size throughout the paligenotic cascade. Average cell area computed with ImageJ/FIJI plotted as a function of time after injury. Black Dots average per mouse. Red line average of three mice per condition.

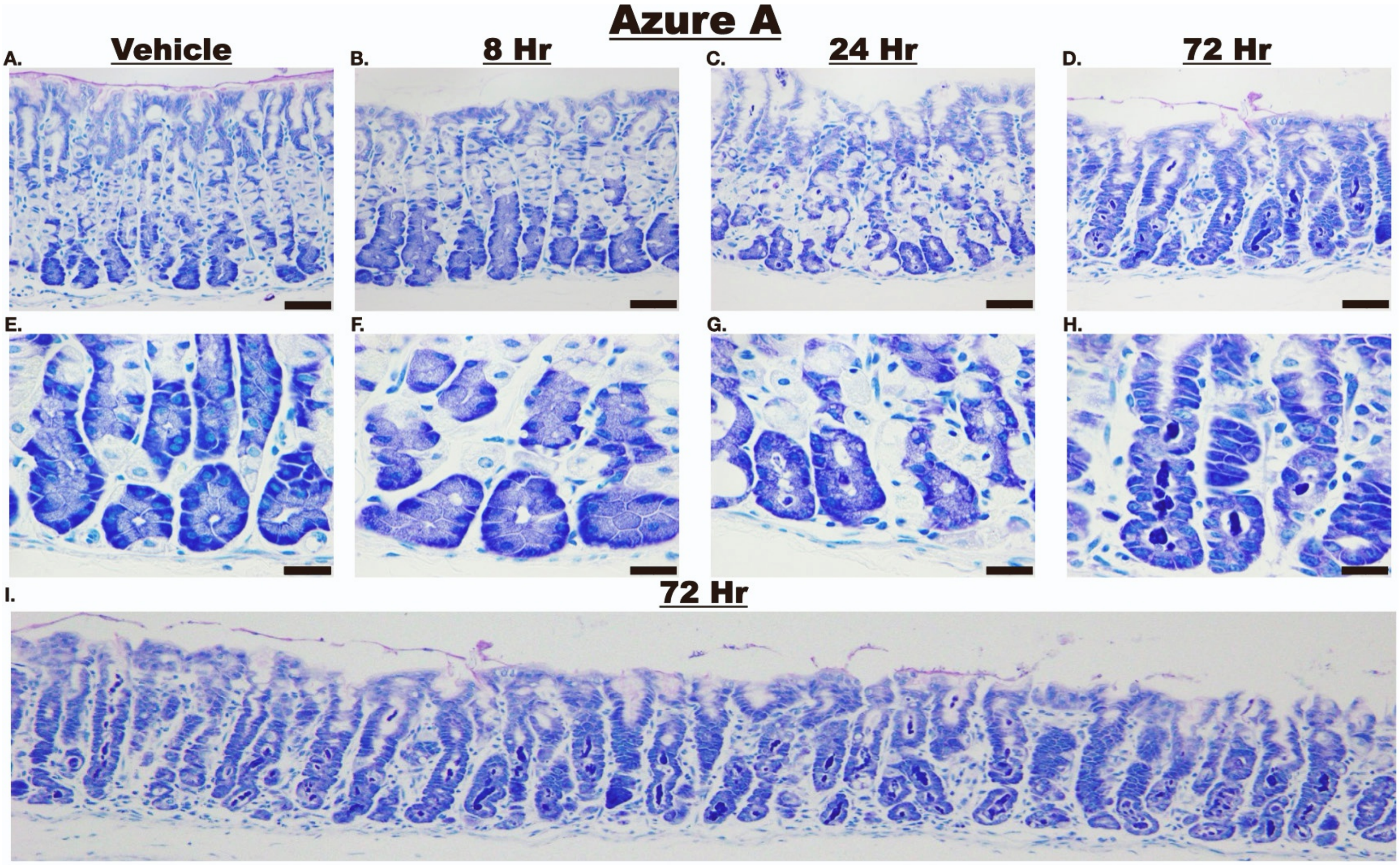

Supplemental Figure 4. **Azure A stains the endoplasmic reticulum of the gastric chief cell and permitted tracking of this compartment during paligenosis.** Azure A histological stain highlights subcellular changes occurring within chief cells undergoing paligenosis. (**A,E**) Vehicle-treated wild-type mice demonstrated strong dark blue basal staining tapering towards cell apex where zymogenic granules that were not Azure A avid localize. Chief cell staining paralleled confocal microscopy using anti-protein disulfide isomerase, an established endoplasmic reticulum marker PDI (cf. Supplemental Figure 5). Nuclei were royal (light?) blue due to affinity of Azure A for deoxyribonucleic acids (Supplemental Figure 7). (**B,F**) At 8 hours after injury the intense dark blue basal staining began to dissipate. (**C,G**) At 24 hours after injury, Azure A reactivity filled gland lumens, and the apical-basal distance became smaller. (**D,H**) At 72 hours after injury, the chief cells were small, cuboidal, and had scant cytoplasm. Gland lumens were full of Azure A-avid material. **I**. The synchronicity of the tamoxifen injury model can be appreciated at 72 hours in a lower-magnification image demonstrating all glands full of Azure A-reactive material. As this material labels with ER marker PDI (cf. Supplemental Figure 5), and Azure A stains ER in homeostasis, the lumen material may largely be extruded ER. Given (**A-C**). Scale bars in A-D = 50 μm; E-H = 20 μm.

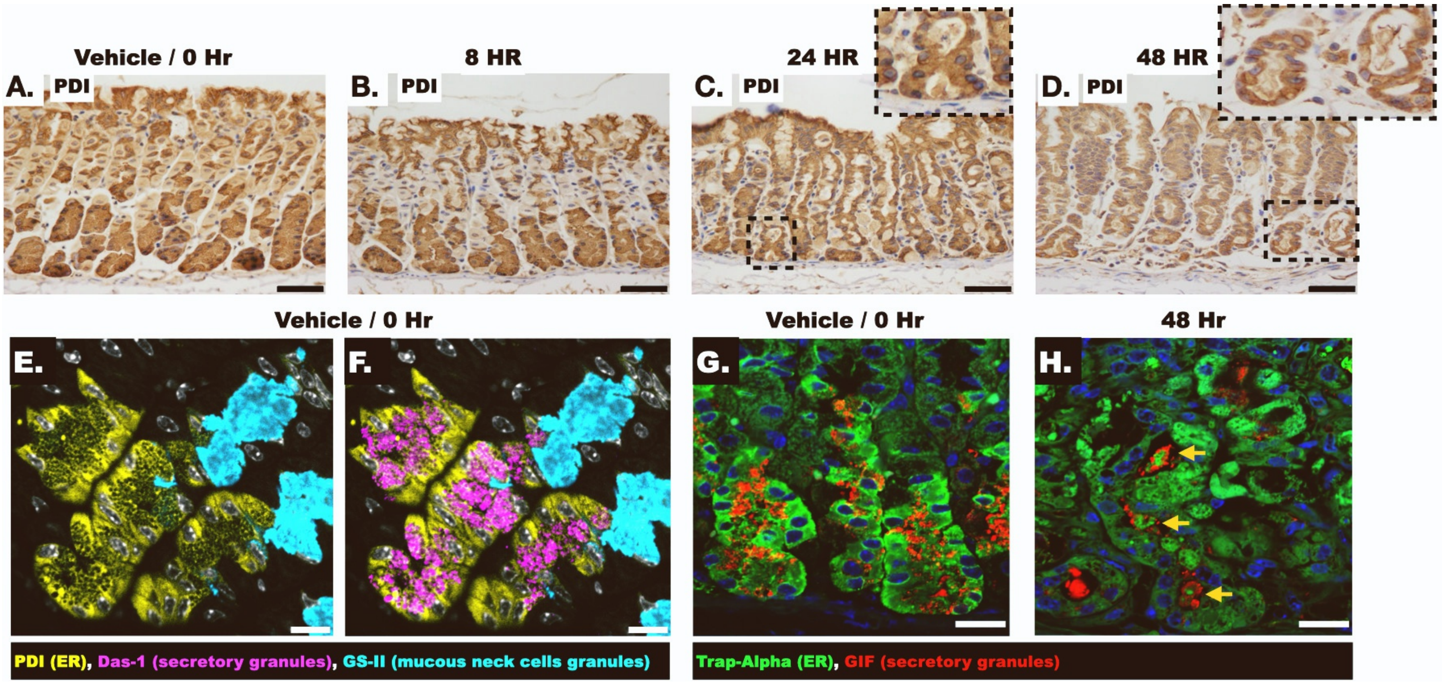

Supplemental Figure 5. Excretion of endoplasmic reticulum markers. IHC during paligenosis (**A**. vehicle, **B**. 8 hr, **C**. 24 hr, **D**. 72 hr) demonstrates secretion of PDI. **E, F**. The Gastric Chief Cell Cytoplasm was primarily composed of Endoplasmic Reticulum (PDI, Yellow) and Zymogenic Granules (Das-1, Magenta). Epitopes marked by these two antibodies are mutually exclusive. The lectin Griffonia simplifolia (GSII) was used as a neck cell marker (cyan). G-H. Epitopes from the ER as well as the secretory granules are excreted following injury. G. Vehicle treated. H. 24 hours after injury. Yellow arrows: highlight extracellular/luminal Trap-Alpha. Scale bars in A-D = 50 µm, Scale bars in E-H = 20 µm.

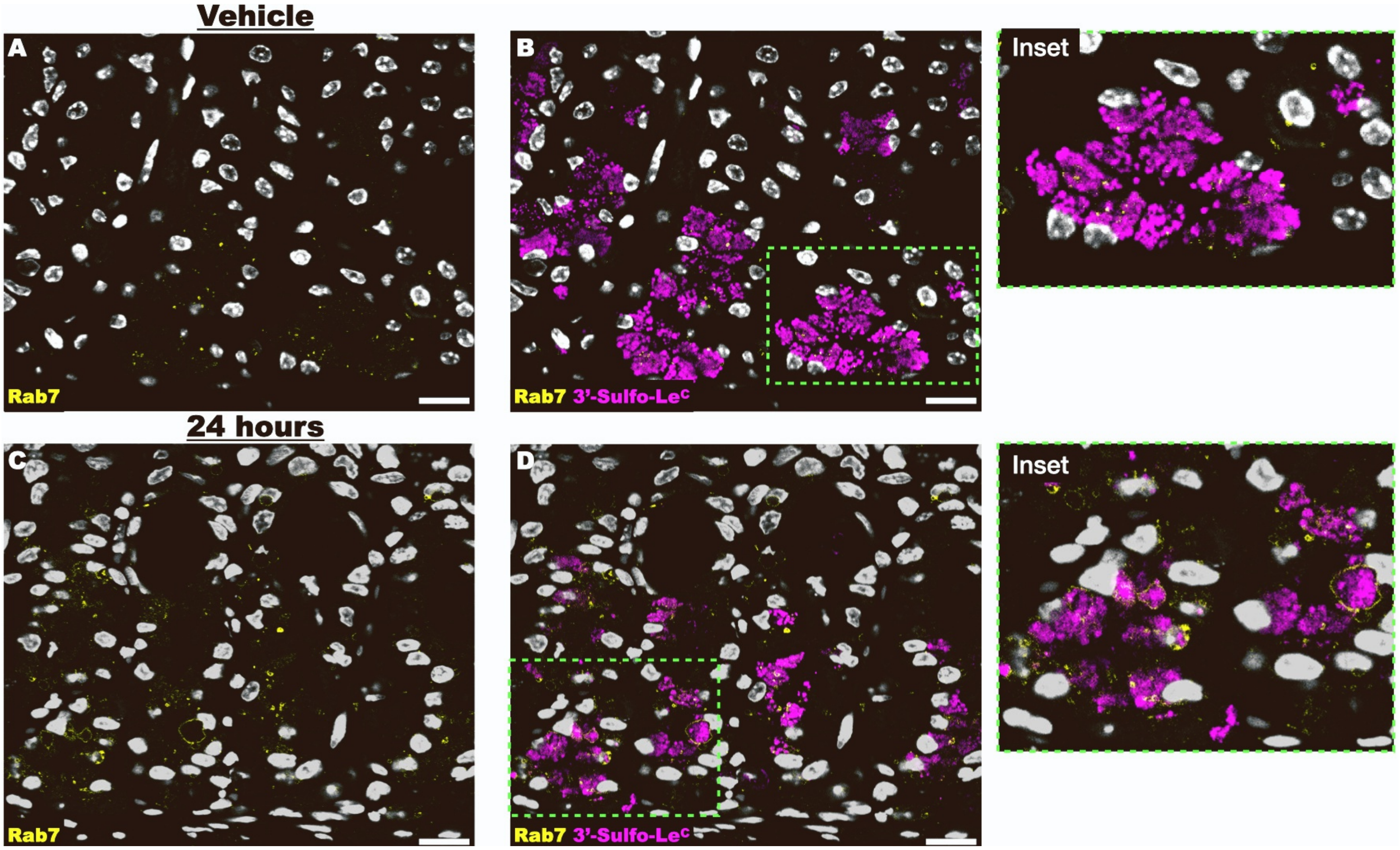

Supplemental Figure 6. A portion of the sulfated mucins are enveloped by vesicular structures decorated with RAB7. A, B. Vehicle-treated stomach demonstrates scattered, small RAB7 positive vesicles, interspersed among zymogenic granules (RAB7 is typically a marker of late endosomes and lysosomes). **C,D**. 24 hours after injury, large RAB7-decorated vesicles envelope sulfated mucins. Pseudocoloring: White: Nucleus (DAPI); Yellow: RAB7; Magenta: 3'-Sulfo-Le^{C} (Das-1). Scale bars = 20 μm.

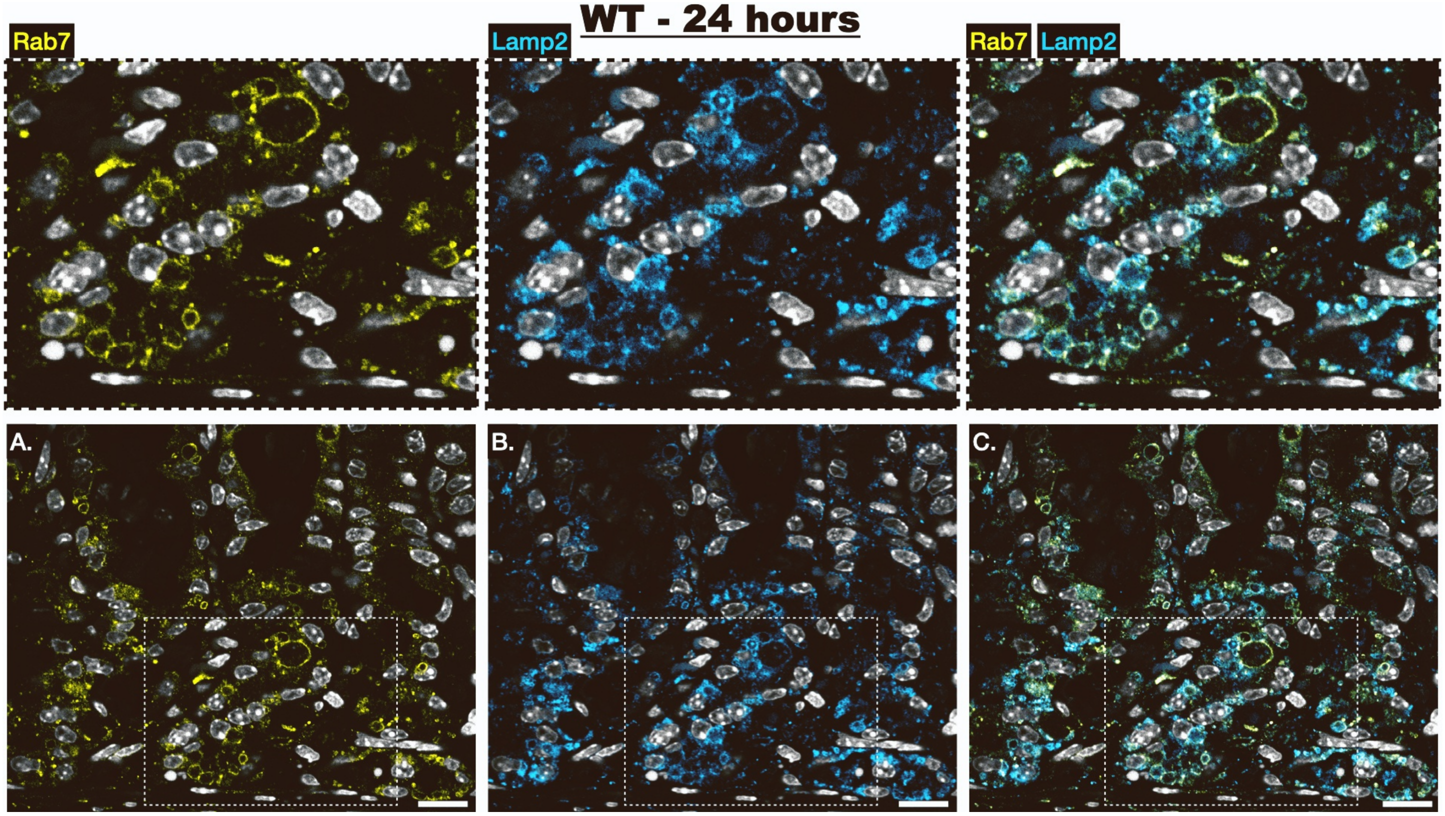

Supplemental Figure 7. RAB7 (yellow) and LAMP2 (cyan) colocalized on large intracellular vesicles 24 hours after injury in wild-type C57/Black6 mice. Nuclei (via DAPI) are white. Scale bars = 20 µm.

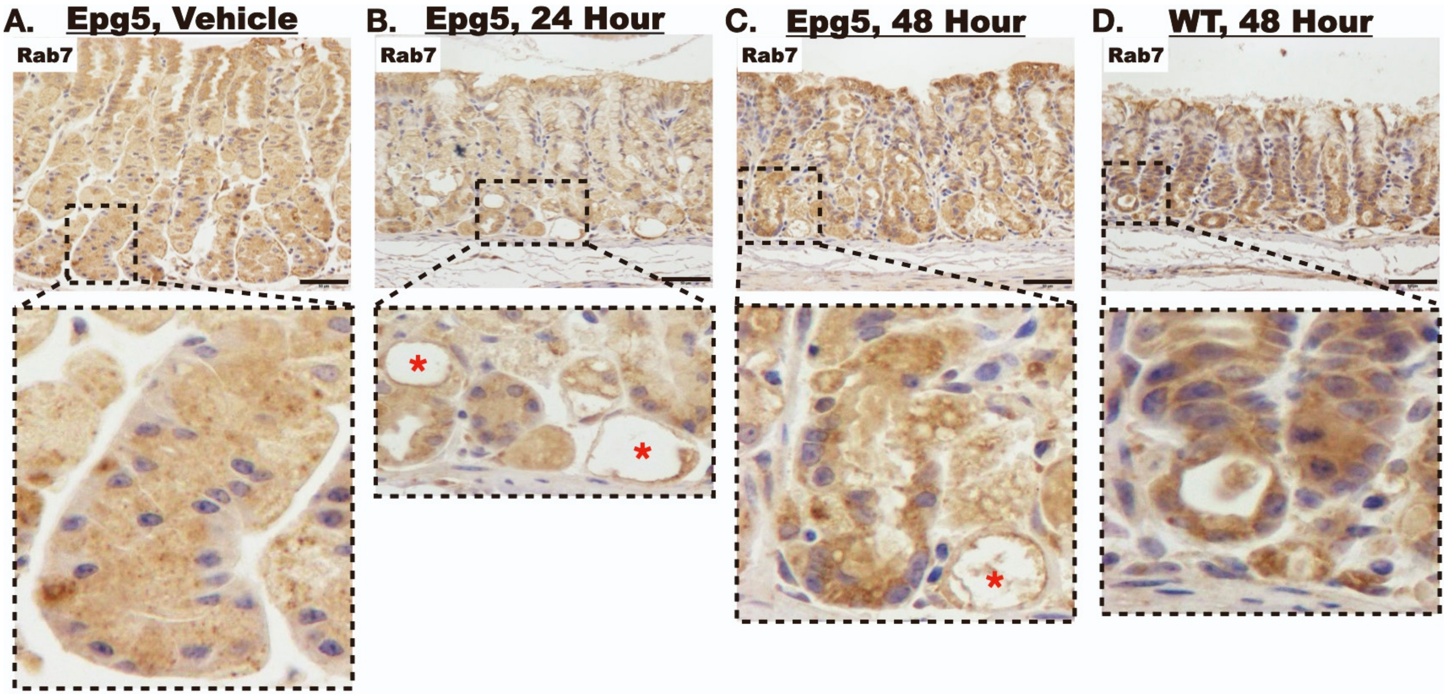

Supplemental Figure 8. Distribution of RAB7 in *Epg5* null mice during paligenosis. A. In *Epg5*$^{-/-}$ mice, RAB7 was absent from the apical membrane and formed small punctae throughout the cell. This is similar to what was observed in wild-type C57/Bl6 mice (cf Fig. 5A). **B.** RAB7 associated with the apical membrane at 24 hours after injury in the *Epg5* null background, but not in wild-type mice (**D**). **C**. At 48 hours a few glands are still composed of very flat chief cells and larger Rab7 structures are appreciated at this time point compared to (**D.**) Wild-Type stomachs 48 hours after injury. Scale bars = 50 μm.

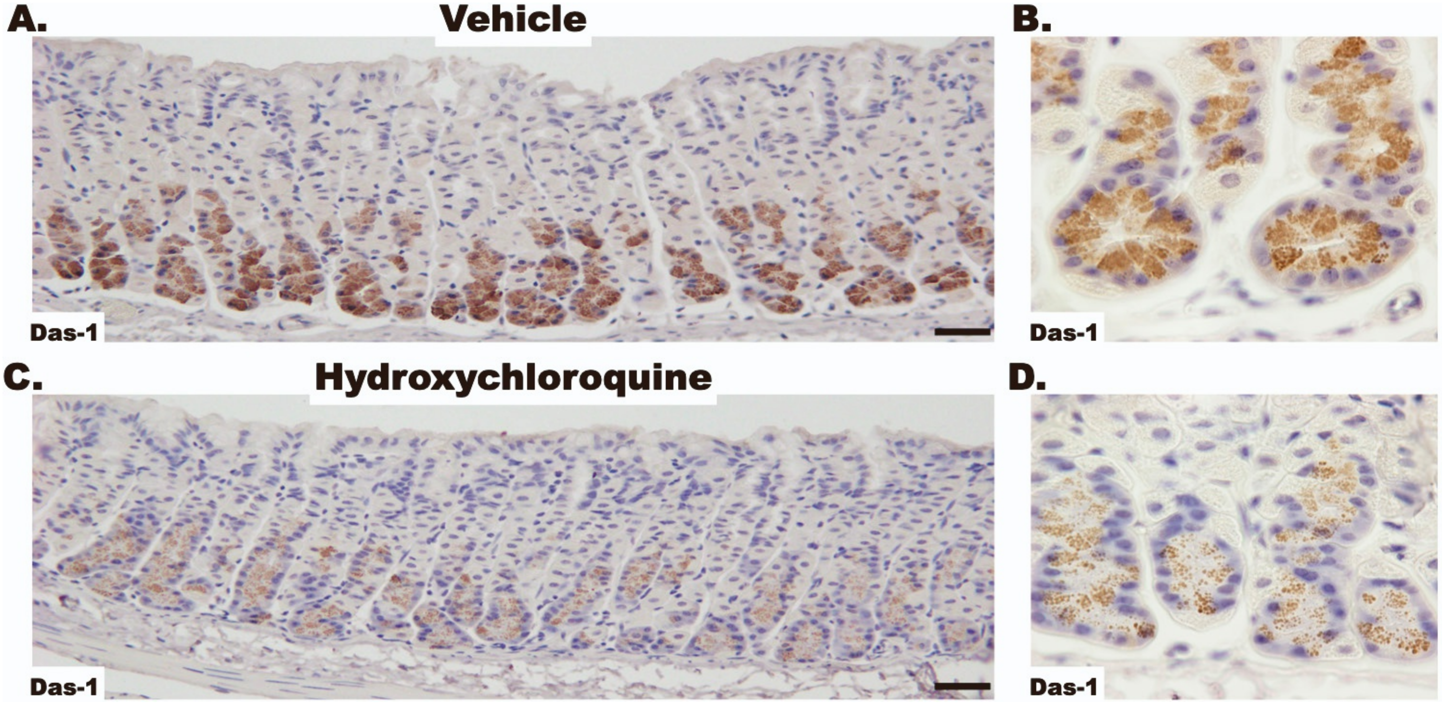

Supplemental Figure 9. **Hydroxychloroquine treatment results in smaller zymogenic granules.** **A.** Wild-type, vehicle treated mice. **B.** High-power view wild-type, vehicle treated mouse stomach. **C.** Hydroxychloroquine treated mouse stomach. **D.** High-power view hydroxychloroquine treated mouse stomach. IHC performed with the Das-1 antibody, which is reactive against 3'-Sulfo-Le^{C} in murine tissue. **Scale** Bars = 50 μm.

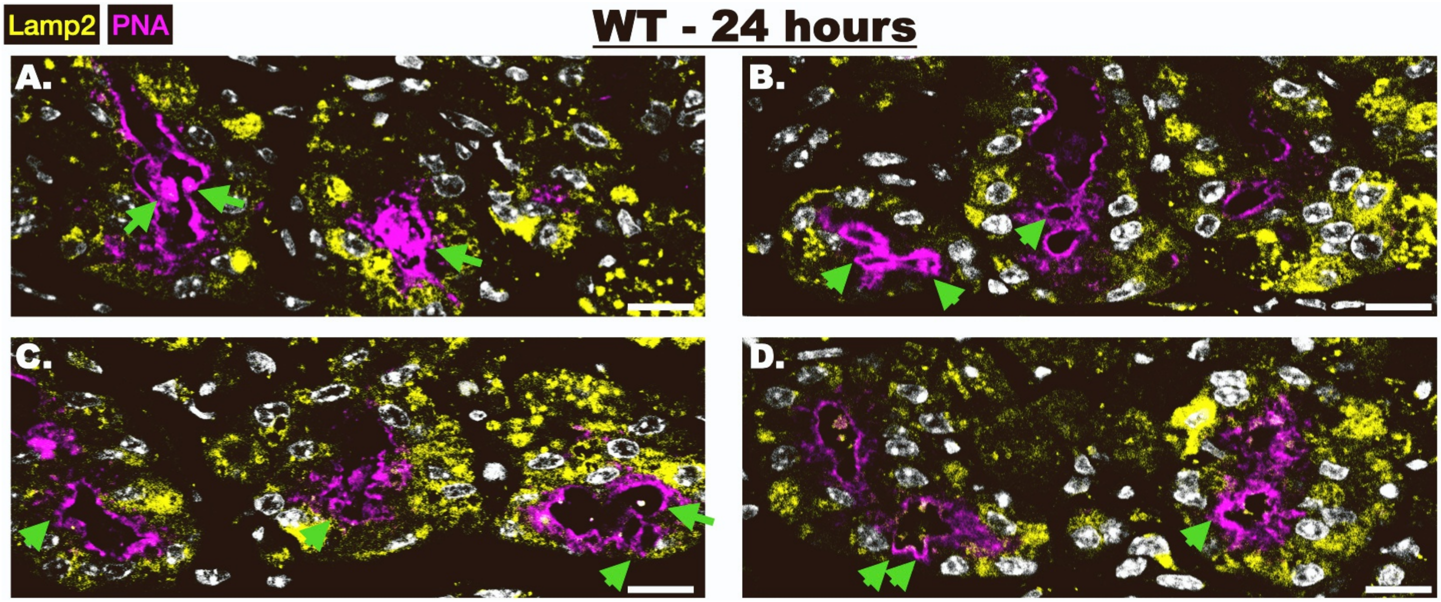

Supplemental Figure 10. The dramatic apical deformities are present at 24 hours following injury in hydroxychloroquine treated animals. A-D. 4, representative confocal micrographs of tamoxifen-injured hydroxychloroquine treated mice demonstrates identical invaginations (green arrowheads) and membrane flaps (green arrows). Similar to wild-type Mice, and unlike what occurs in *Epg5*$^{-/-}$ mice, there is little overlap of lysosomal markers (LAMP2) and membrane markers (PNA) in hydroxychloroquine treated mice. Pseudocoloring: White: DAPI/Nuclear; Yellow: Lamp2; Magenta: PNA Lectin. Scale bars = 20 µm.

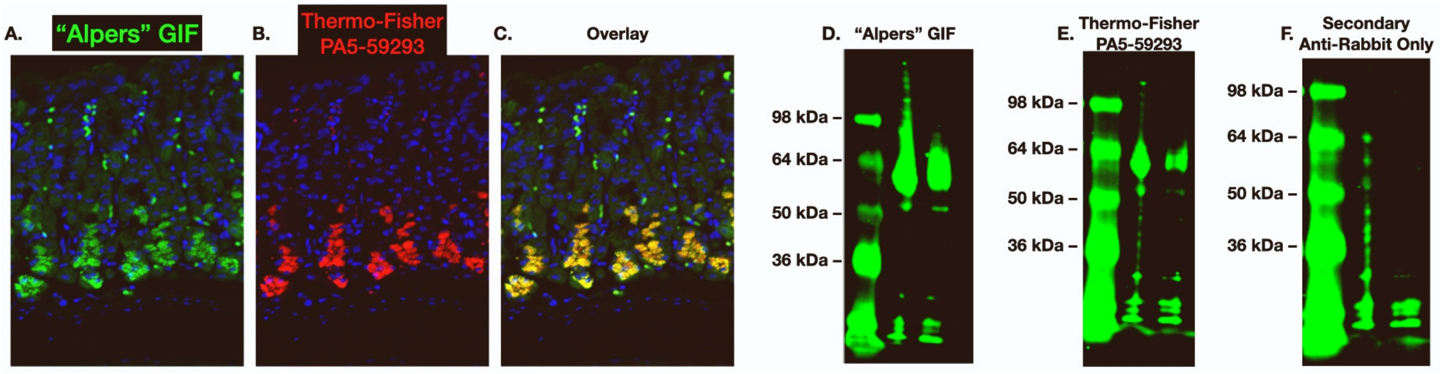

Supplemental Figure 11. Validation that the Thermofisher Anti-GIF antibody PA5-59293 is reactive against murine GIF as well. A-C. Identical histologic distribution of PA5-59293 (red) and anti-GIF (green, produced by David Alpers). **D-F.** Western blot mouse stomach lysate with vehicle treatment and 24 hours after injury demonstrating comparable bands. The confusion may derive from GIF being present human parietal cells, but murine chief cells. Nonetheless, the antibody PA5-59293 can be used in mouse tissue.
